# A novel interleukin-2-based fusion molecule, HCW9302, differentially promotes regulatory T cell expansion to treat atherosclerosis in mice

**DOI:** 10.3389/fimmu.2023.1114802

**Published:** 2023-01-25

**Authors:** Xiaoyun Zhu, Qiongzhen Li, Varghese George, Catherine Spanoudis, Crystal Gilkes, Niraj Shrestha, Bai Liu, Lin Kong, Lijing You, Christian Echeverri, Liying Li, Zheng Wang, Pallavi Chaturvedi, Gabriela J. Muniz, Jack O. Egan, Peter R. Rhode, Hing C. Wong

**Affiliations:** HCW Biologics Inc., Miramar, FL, United States

**Keywords:** IL-2, IL-2-based fusion molecule, Tregs, M2 macrophages, myeloid-derived suppressor cells, inflammatory diseases, atherosclerosis

## Abstract

Atherosclerosis is a chronic inflammatory disease caused by deposition of oxidative low-density lipoprotein (LDL) in the arterial intima which triggers the innate immune response through myeloid cells such as macrophages. Regulatory T cells (Tregs) play an important role in controlling the progression or regression of atherosclerosis by resolving macrophage-mediated inflammatory functions. Interleukin-2 (IL-2) signaling is essential for homeostasis of Tregs. Since recombinant IL-2 has an unfavorable pharmacokinetic profile limiting its therapeutic use, we constructed a fusion protein, designated HCW9302, containing two IL-2 domains linked by an extracellular tissue factor domain. We found that HCW9302 exhibited a longer serum half-life with an approximately 1000-fold higher affinity for the IL-2Rα than IL-2. HCW9302 could be administered to mice at a dosing range that expanded and activated Tregs but not CD4^+^ effector T cells. In an *ApoE^-/-^* mouse model, HCW9302 treatment curtailed the progression of atherosclerosis through Treg activation and expansion, M2 macrophage polarization and myeloid-derived suppressor cell induction. HCW9302 treatment also lessened inflammatory responses in the aorta. Thus, HCW9302 is a potential therapeutic agent to expand and activate Tregs for treatment of inflammatory and autoimmune diseases.

## Introduction

Atherosclerosis is a chronic inflammatory disease. Atherosclerotic plaque formation in the arterial wall is initiated by retention of oxidized LDL in the arterial intima. This leads to activation of endothelial cells, and recruitment and migration of monocytes across the endothelial barrier (1). These recruited monocytes subsequently differentiate into macrophages which phagocytose and degrade the accumulated oxidized LDL (1). Eventually, the macrophage metabolization pathways become overloaded, which lead to lipid accumulation and dysfunction of the lysosomal compartment (2). Consequently, macrophages transform into foam cells with impaired lipid metabolization that secrete proinflammatory factors which further promote plaque development (2). Thus, the accumulation of oxidized LDL to levels that overcome the metabolic capacity of macrophages and dysfunctional lysosomes of foam cells trigger the process of atherogenesis (3, 4).

Decreased Treg numbers and/or impaired Treg immunosuppressive functions were reported to promote atherosclerosis in animal and human studies (5–7). Depletion of Foxp3^+^ Tregs exacerbated atherosclerosis and was also associated with higher plasma levels of atherogenic lipoprotein in *Ldlr^-/-^* mice (8, 9). It has also been shown that infusion of purified spleen Tregs reduced atherosclerosis and induced a more stable plaque phenotype in *ApoE^-/-^* mice (6). In human atherosclerotic plaques, Tregs are largely absent during all stages of development, with less than 5% of infiltrating T cells being Foxp3^+^ (7). Moreover, Treg numbers have been shown to be lower in vulnerable compared to stable plaques (10). The strong anti-atherogenic activities of Tregs are linked through the production of anti-inflammatory cytokines IL-10 and TGF-β in murine models (11, 12). Tregs prevent T cell polarization into proinflammatory Th1 and Th17 subtypes and limit their pathogenic activities. They also inhibit the proinflammatory properties of macrophages and shift macrophage differentiation toward an anti-inflammatory phenotype (13). Previous studies have shown that foam cell formation was inhibited when Tregs were co-cultured with macrophages which downregulated CD36 and the class A scavenger receptor known to be involved in initiation of atherosclerotic lesions (13, 14). Tregs also enhance macrophage efferocytosis by inducing IL-13 and IL-10 production (15, 16). Endothelial cell activation and leukocyte recruitment can also be regulated by Tregs, independent of their immunosuppressive activities on T cells. Monocyte recruitment into atherosclerotic lesions is impeded through the inhibition of MCP-1 expression in dendritic cells and macrophages (9).

IL-2, a common gamma chain cytokine, is essential for Treg homeostasis *in vivo* (17). Therefore, recombinant human IL-2 (rhIL-2) has been explored to expand Tregs *in vivo* to treat inflammatory diseases (13). In patients with chronic graft-versus-host disease, low-dose IL-2 administration results in increased levels of circulating Tregs without changing effector T cell levels (18). A substantial proportion of these treated patients showed clinical benefit. In patients with hepatitis C virus-induced autoimmune vasculitis, IL-2 treatment was also able to increase the percentage of circulating Foxp3^+^ Tregs and improve clinical symptoms (19). Similarly, clinical benefit of low-dose IL-2 treatment was observed in patients with type 1 diabetes (20). For atherosclerosis, local delivery of IL-2 to atherosclerotic lesions or treatment with anti-CD3 and IL-2/anti-IL-2 mAb complexes led to a reduction in atherosclerosis due to Treg expansion in *ApoE^-/-^* mice (21, 22). These findings are being translated into clinical applications, with the safety and efficacy of low-dose IL-2 being assessed in double blind, placebo-controlled phase I/II clinical trials (LILACS, IVORY) in patients with stable ischemic heart disease and acute coronary syndromes (23, 24). However, rhIL-2 long-term therapeutic success in expanding Tregs in the clinic has been restricted by its rapid clearance from circulation and potential for inducing cytokine release syndrome-related toxicities (25, 26). We recently described a protein expression platform based on the extracellular domain of human tissue factor (TF) protein as a scaffold to create novel multi-functional fusion molecules with improved pharmacokinetic (PK) and activity profiles (27–29). We employed this technology to construct a fusion protein, designated HCW9302, containing two IL-2 domains linked by a TF domain, to extend the half-live of IL-2 for Treg expansion. In this study, we showed that HCW9302 has a much longer serum half-life and higher affinity with IL-2Rα than rhIL-2. HCW9302 was found to preferentially activate and expand Tregs both *in vitro* and *in vivo*. The progression of atherosclerosis in both *ApoE^-/-^* and *Ldlr^-/-^* mice was suppressed by subcutaneous administration of HCW9302. These effects were mediated through the mechanism of activating and expanding Tregs as well as potentially HCW9302-induced M2 macrophages and myeloid derived suppressor cells (MDSCs) to control inflammation and atherosclerosis.

## Materials and methods

### Construction and production of HCW9302 fusion protein

**Figure 1:**
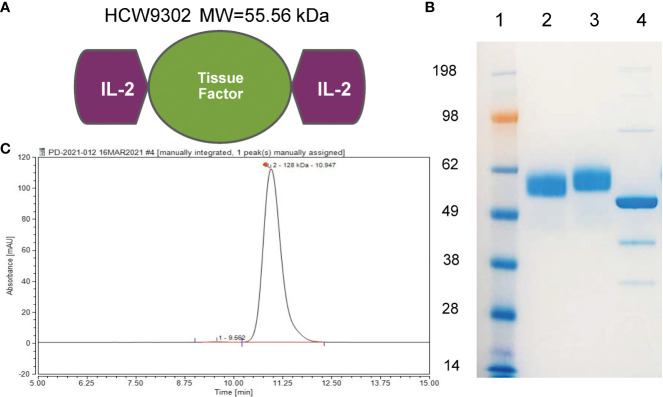
Characterization of HCW9302. **(A)** A schematic model of HCW9302 protein comprising two IL-2 domains linked by an extracellular tissue factor domain. **(B)** The polypeptides of HCW9302 are highly glycosylated. To examine the molecular weight characteristics, deglycosylated and non-deglycosylated protein samples were analyzed on 4%–12% SDS-PAGE Bis-Tris gels under denaturing conditions and stained with InstantBlue. Lane 1, SeeBlue Plus2 Pre-stained Standard; Lane 2, non-deglycosylated, non-reduced HCW9302; Lane 3, non-deglycosylated, reduced HCW9302; Lane 4, deglycosylated, reduced HCW9302. **(C)** High-performance liquid chromatography (HPLC)-SEC profile of purified native HCW9302 samples.

HCW9302 is a recombinant fusion protein, constructed by fusing one human IL-2 domain to the N-terminus of the soluble extracellular domain of human tissue factor, and another human IL-2 domain to the C-terminus of the human tissue factor domain (**Figure 1A**). The corresponding coding DNA sequences were synthesized (Genewiz), cloned into pMSGV-1 modified expression vectors (30) and transfected into CHO.K1 cells (ATCC, CCL-61). HCW9302 was expressed in CHO cells and secreted into the culture media. HCW9302 expression was then detected with product specific ELISA formats using an anti-human tissue factor antibody (HCW Biologics, HCW9101) for capture and an anti-human IL-2 antibody (R&D Systems, BAF202) for detection. Production cell banks for HCW9302 were generated from stably transfected clonal cell lines following limiting dilution cloning. Subsequent fusion protein production was conducted using fed-batch methods with chemically defined media in shake flasks or stir tank bioreactors. HCW9302 was purified from clarified culture media using immunoaffinity chromatography with anti-TF Ab-conjugated Sepharose resin, and then buffer-exchanged into PBS. A GMP-suitable manufacturing process (scaled from 2 L to 200 L) was developed for HCW9302 consisting of immunoaffinity chromatography, low pH viral inactivation/depth filtration, multimodal chromatography, nanofiltration, and ultrafiltration/diafiltration steps employing commercially scalable methods. The purified HCW9302 product was characterized and released using qualified test methods per established specifications.

### Surface plasmon resonance

Surface plasmon resonance was performed by Acro Biosystems (Newark, DE). Binding affinities of HCW9302 and human IL-2 (Acro, S13-H5113) were measured by surface plasmon resonance on human IL-2Rαβγ, IL-2Rβγ, or IL-2Rα on a Biacore 8K (Cytiva). The Fc-tagged human IL-2Rαβγ (Acro, ILG-H5257), IL-2Rβγ (Acro, ILG-H5254), or IL-2Rα (Acro, ILA-H5251) were captured by immobilized anti-human IgG (Fc) antibody (Cytiva, 29234600) on CM5 chips (Cytiva, BR100530). To measure binding to IL-2Rs, 2-fold dilutions of HCW9302 (0.061-0.977 nM) or IL-2 (0.061-3.906 nM) for IL-2Rαβγ binding, of HCW9302 (0.488-31.25 nM) or IL-2 (0.488-31.25 nM) for IL-2Rβγ binding, and of HCW9302 (0.049-3.125 nM) or IL-2 (0.195-25 nM) for IL-2Rα binding, were injected over the chip surface for 120 seconds and dissociations were monitored for 300 seconds. The surface was regenerated after each injection by washing with 3 M magnesium chloride for 30 seconds. The binding curves were fitted using a 1:1 fitting model. To confirm binding affinities of HCW9302 and human IL-2 on IL-2Rα, his-tagged human (Acro, ILA-H5251), mouse (Acro, ILG-M52H9), or cynomolgus (Sino, 90265-C08H) IL-2Rα were chemically immobilized by amine coupling on CM5 chips. Two-fold dilutions of HCW9302 (0.195-6.25 nM) or IL-2 (0.195-25 nM) for human IL-2Rα binding, of HCW9302 (0.195-25 nM) or IL-2 (0.195-25 nM) for mouse IL-2Rα binding, and of HCW9302 (0.195-6.25 nM) or IL-2 (0.195-25 nM) for cynomolgus IL-2Rα binding, were injected respectively over the chip surface for 90 seconds and dissociations were monitored for 210 seconds. The surface was regenerated after each injection by washing with 3 M magnesium chloride for 30 seconds. The binding curves were fitted using a 1:1 fitting model.

### *In vitro* verification of HCW9302 activity

CTLL-2 (ATCC, TIB-214) and 32Dβ cells (31) were maintained in IMDM supplemented with 10% FBS and 25 ng/ml of human recombinant IL-2 (Peprotech, 200-02). For cell assays, CTLL-2 and 32Dβ cells were washed 5 times and seeded at 2 x 10^4^ cells/well in 96-well plates in IMDM with 10% FBS. Purified HCW9302 was added to the wells at 1:3 serial dilutions and cells were incubated for 72 hours at 37°C in a CO_2_ incubator. PrestoBlue proliferation reagent (ThermoFisher, A13261, 20 µL/well) was added. After 4 hours, absorbance was measured at 570/610 nm to determine cell proliferation based on reduction of the PrestoBlue™ reagent to resorufin by metabolically active cells. The bioactivity of human recombinant IL-2 (aldesleukin, NDC 65483-116-07, or Peprotech, 200-02) was assessed as a positive control.

Peripheral blood mononuclear cells (PBMC) from healthy donors were isolated from whole blood buffy coats (Continental Services Group, Miami, FL) by Ficoll Paque Plus (Millipore/Sigma, GE17144003). PBMCs were treated with ammonium chloride-potassium (ACK) lysing buffer (Thermo Fisher Scientific, A1049201) to remove red blood cells. Cells were washed with IMDM-10% FBS and counted. Cells (1.8 x10^6^ in 100 µl/tube) were seeded in flow tubes and incubated with 50 μl of l/10-diluted HCW9302 or IL-2 (Proleukin^®^ (aldesleukin), NDC 65483-116-07) (15000, 1500, 150, 15, 1.5, 0.15, or 0 pM) and 50 µl of anti-CD8-BV605 (BioLegend, 344742; 1:50). Cells were incubated for 30 min at 37° C. Pre-warmed BD Phosflow Fix Buffer I (200 µl) (BD Biosciences, 557870) was added for 10 min at 37° C to stop the stimulation. Cells (4.5 x10^5^ cells/100 µl) were transferred to V-shape 96-well plates and were spun down followed by permeabilization with 100 µl of pre-cooled BD Phosflow Perm Buffer III (BD Biosciences, 558050) for 30 min on ice. Cells were washed twice with 200 µl of FACS buffer and stained with a panel of fluorescent antibodies: anti-CD25-PE (BD Biosciences, 555432; 1:100), CD4-PerCP-Cy5.5 (BD Biosciences, 560650; 1:50), CD56-BV421 (BioLegend, 362552; 1:100), and pSTAT5a-AF488 (BD Biosciences, 612598; 1:100), to distinguish different lymphocyte subpopulations and pSTAT5a status. Cells were spun down and resuspended in 200 µl of FACS buffer for FACSCelesta analysis.

Human CD4^+^CD127^low^CD25^+^ Tregs and CD4^+^CD25^-^ Tresp cells were isolated from fresh PBMCs of healthy donors using EasySep™ Human CD4^+^CD127^low^CD25^+^ Regulatory T Cell Isolation Kit (Stemcell, 18063). Tregs were expanded in RPMI-1640 supplemented with 10% FBS (R10), Dynabeads Human T-activator CD3/CD28 (ThermoFisher, 11131D) at a bead to total cell ratio of 4:1, and 50 nM HCW9302. Cells were counted and stained to check purity at days 1, 5, 10, and 15. Functionality of human Treg product was assessed on the basis of the ability to suppress proliferation of autologous CD4^+^CD25^−^ Tresp cells as described (32). Briefly, cryo-stored Tresp cells from the same donors were thawed, washed in turn with 10 ml R10 and PBS. Tresp cells were stained with CellTrace Violet cell proliferation kit (ThermoFisher, C34557) at 1/1000 dilution in 1 ml of PBS. After incubation for 20 min at 37° C, 5 ml of R10 was added, and cells were incubated for an additional 5 min. The cells were centrifuged, resuspended in R10 and analyzed by flow cytometry to confirm that the cells were labeled. Labeled Tresp cells were washed once in 10 ml of cold R10 and resuspended in prewarmed R10 for plating. Tregs were seeded in a round bottom 96 well cell culture plate to reach Treg : Tresp ratios of 1:32, 1:16, 1:8, 1:4, 1:2, 1:1, and 1:0 in a volume of 100 µl. Labeled Tresp cells (1 × 10^5^) were added to the plate and Dynabeads Human T-activator CD3/CD28 (ThermoFisher, 11131D) were added at a bead to total cell ratio of 1:75. In order to determine maximum proliferation of Tresp cells, Tresp cells were cultured with or without beads in the absence of Tregs. The final volume was adjusted to 200 µl for all conditions. Plates were incubated at 37° C for 5 days. On day 5, the plates were spun down and the individual wells were harvested. Proliferating Tresp cells were defined as the percentage of CellTrace Violet (BV421)^+^ cells shifted from the original Tresp population. The mean inhibition of proliferation (% suppression) found at the different Treg : Tresp ratios was calculated as (Proliferation_Tresp only_-Proliferation_Tresp with Treg_)/Proliferation_Tresp only_ × 100. A nonlinear fit of the percentage of suppression vs. the number of added Treg for each condition was calculated using GraphPad Prism 9.

### Animals and experimental protocols

Six-week-old female C57BL/6J (strain# 000664), B6. *ApoE* deficient mice (B6.129P2-*Apoe^tm1Unc^*/J, strain# 002052), or B6. *Ldlr* deficient mice (B6.129S7-*Ldlr^tm1Her^*/J, strain# 002207), were purchased from the Jackson Laboratory (Bar Harbor, ME). For atherosclerosis studies, *ApoE* deficient mice or *Ldlr* deficient mice were maintained on a high fat Western diet (TD.88137, Envigo). A group of *Ldlr*-deficient mice was also fed the regular chow diet. Six weeks later the mice were administered HCW9302 subcutaneously at 3 mg/kg. The mice received 2 additional subcutaneous doses of HCW9302 at weeks 9 and 12 while continuing on the Western diet. Control mice received subcutaneous PBS. Mice were euthanized at 20 weeks of age (14 weeks after initiating the Western diet) and aortic atherosclerotic lesions were assessed.

To evaluate the pharmacokinetic profile of HCW9302, female C57BL/6J mice (three mice/time point) were subcutaneously injected with HCW9302 (3 mg/kg) and blood was collected at various time points from 2 to 24 h post injection. Serum concentrations of HCW9302 were evaluated using ELISA formats with an anti-human tissue factor antibody (HCW Biologics, HCW9101) for capture and an anti-human IL-2 antibody (R&D Systems, BAF202) for detection. HCW9302 levels were fit with a one-compartment model using PK Solutions 2.0 (Summit Research Services, Montrose, CO).

To evaluate the effect of HCW9302 on plasma cytokines, blood was collected through submandibular vein puncture one week after the 3^rd^ dose of HCW9302. Plasma was collected and run at a 2-fold dilution with PBS, and analyzed at Eve Technologies (Calgary, Canada) using Mouse Cytokine Proinflammatory Focused 10-Plex Discovery Assay Array (Eve Technologies, MDF10).

### Enface analysis of aortic lesions

Euthanized *ApoE* deficient and *Ldlr* deficient mice were perfused through the left ventricle with 4% paraformaldehyde-sucrose followed by PBS-EDTA. The whole aorta including heart was harvested and fixed in 4% paraformaldehyde-sucrose. The soft/loose perivascular adipose tissue was gently removed from around the aorta from heart to iliac bifurcation. The branching arteries and heart were removed under the dissection microscope at 20-25x magnification using fine iris scissors and delicate forceps. The whole aorta was split longitudinally, pinned in the black wax petri dish, fixed overnight with 4% paraformaldehyde-sucrose at room temperature, rinsed, fixed with 70% ethanol, and stained using Sudan IV staining solution to identify the plaques as described (33). The total aorta surface area and plaque surface area stained with Sudan IV was captured by a dissection microscope (AmScope, Feasterville, PA, USA). Quantification of the Sudan IV-stained lipid rich plaque area was done using Image J software (https://www.fiji.sc/).

### Aorta collection and aortic sinus sectioning

Euthanized *ApoE* deficient mice were perfused through the left ventricle with cold PBS-EDTA. The whole aorta including heart was harvested. The aorta including parts of arch, thorax, and abdomen were collected for flow cytometric analysis, qPCR, or RNA-seq. The heart was cut so that all three aortic valves were in the same geometric plane. The upper portion of the heart was embedded in O.C.T., frozen in the Peltier stage of the cryostat (Leica CM1950 Cryostat) and processed for sectioning. Sections (10 μm) were collected onto Fisher Superfrost Plus-coated slides, starting from where the aorta exits the ventricle and moving towards the aortic sinus over ~650–700 μm. Sections were collected following the scheme as described (34), each slide (at least 5 sections per mouse) contained 9–12 aortic root sections collected at 40 μm intervals; with this scheme, consecutive, or immediately adjacent sections which are morphologically and compositionally identical are located in separate slides, allowing for different staining methods to be conducted. Additional sections were collected at the end to be used as controls in immunostaining procedures. Lesion analysis with hematoxylin and eosin (H&E) staining was conducted as described previously (34). The images of lesions were taken with a Zeiss Axio Imager 2 microscope and analyzed by calculated aortic surface area covered by lesions using Image J software.

### Immunohistochemistry

For immunostaining, cryosections on slides were fixed with cold acetone, permeabilized with 0.05% Triton X-100, and blocked with 5% normal goat serum. The sections were incubated with antibodies against Foxp3 (Thermo Fisher, 14-5773-82; 1:50) to detect Treg cells, CD68 (Bio-Rad, MCA1957; 1:500) to detect macrophages, CD206 (Bio-Rad, MCA2235; 1:500) to detect M2 macrophages, Ly5g+Ly5c (Abcam, ab25377; 1:100) to detect MDSCs, and arginase 1 (Thermo Fisher, PA5-85267; 1:100) to detect M2 macrophages and MDSCs. Sections were then incubated with appropriate secondary antibodies and detected with 3,3’-diaminobenzidine. The slides were counterstained with Mayer’s hematoxylin. To distinguish bona fide target staining from background, the secondary antibody only was used as a control. The images were taken with a Zeiss Axio Imager 2 microscope and analyzed using Image J software.

### Flow cytometric analysis

To evaluate blood lymphocyte subsets, mouse blood samples were collected through submandibular vein puncture and incubated with ACK lysing buffer (Thermo Fisher, A1049201) at 37°C for 5 minutes. To evaluate aortic lymphocyte subsets, whole aortas were digested using a cocktail of Roche liberaseTH (4 U/ml) (Millipore/Sigma, 5401135001), deoxyribonuclease (DNase) I (0.1 mg/ml) (Millipore/Sigma, DN25), and hyaluronidase (60 U/ml) (Millipore/Sigma, H3506) in 1 mol/L CaCl_2_ at 37°C for 15 min. The digested tissue was passed through a 70 μm cell strainer, washed with 1× cold PBS and centrifuged at 350*g* for 10 minutes at 4°C. Lymphocytes were first stained with live/dead cell fixable violet dead cell stain kit (Thermo Fisher, L34955; 1:100) and then blocked with TruStain FcX (anti-mouse CD16/32, BioLegend, 101320; 1:100). The cells were then stained with the following antibodies: anti-CD45 – BV605 (BioLegend, 103140; 1:100), anti-CD3 –PE/Cy7 (BioLegend, 100220; 1:100), anti-CD8a – PerCP/Cy5.5 (BioLegend, 100734; 1:100), anti-CD4 – BV510 (BioLegend, 100559; 1:100), anti-NK1.1 – APC (BioLegend, 108710; 1:100), anti-F4/80 – APC/Cy7 (BioLegend, 123118; 1:100), anti-CD11b – FITC (BioLegend, 101206; 1:100), anti-CD206 – PE (BioLegend, 141704; 1:100), anti-CTLA-4 – BV420 (BioLegend, 106311; 1:100), anti-CD39 – APC (BioLegend, 143810; 1:100), anti-CD25 – APC/Cy7 (BioLegend, 102026; 1:100), anti-Foxp3 – PE (BioLegend, 126404; 1:50), anti-Gata3 – AF488 (BD Bioscience, 560163; 1:50), anti-Tbet – PerCP/Cy5.5 (BioLegend, 644806; 1:50), anti-RORgt – APC (Thermo Fisher, 17-6981-82; 1:50), and anti-Ki67 – AF700 (BioLegend, 652420; 1:50). For Gata3, RORgt, T-bet, Foxp3, and Ki67 staining, the cells were permeabilized using an Invitrogen™ eBioscience™ Foxp3/Transcription Factor Staining Buffer Set (Thermo Fisher, 00-5523), and then stained with anti-Gata3, anti-RORgt, anti-Tbet, anti-Ki67, and anti-Foxp3 antibodies. Flow cytometry analysis was performed on a FACSCelesta (BD Bioscience, Franklin Lakes, NJ, USA) and analyzed using FlowJo software. The antibodies used in this paper are listed in **Supplementary Table 1**.

### RNA isolation and quantitative PCR

Total RNA was extracted from aortas after homogenization using the TRIzol reagent (Invitrogen, Carlsbad, CA, USA) and purified with RNeasy Mini Kit (Qiagen, Germantown, MD, USA). cDNAs were synthesized with the QuantiTect Reverse Transcription Kit (Qiagen, Germantown, MD, USA). Quantitative PCR (qPCR) was performed using a SsoAdvanced™ Universal SYBR^®^ Green Supermix (BioRad, Hercules, CA, USA) and a QuantStudio 3 Real-Time PCR System (Applied Biosystems, Carlsbad, CA, USA) according to the comparative threshold cycle method following manufacturer’s protocol. The amplification reactions were performed in duplicate, and the fluorescence curves were analyzed with the software included with the QuantStudio 3 Real-Time PCR System. 18s RNA was used as an endogenous control reference. The primers used for qPCR are listed in **Supplemental Table 2**.

### RNA-Seq analyses

RNA-seq was performed by Genewiz/Azenta (South Plainfield, NJ, USA). Extracted RNA samples were quantified using a Qubit 2.0 Fluorometer (Life Technologies, Carlsbad, CA, USA) and RNA integrity was checked using an Agilent TapeStation 4200 (Agilent Technologies, Palo Alto, CA, USA). RNA sequencing libraries were prepared using the NEBNext Ultra II RNA Library Prep Kit for Illumina following manufacturer’s instructions (NEB, Ipswich, MA, USA). Briefly, mRNAs were first enriched with Oligo(dT) beads. Enriched mRNAs were fragmented for 15 minutes at 94°C. First strand and second strand cDNAs were subsequently synthesized. cDNA fragments were end repaired and adenylated at 3’ ends, and universal adapters were ligated to cDNA fragments, followed by index addition and library enrichment by limited-cycle PCR. The sequencing libraries were validated on the Agilent TapeStation (Agilent Technologies, Palo Alto, CA, USA), and quantified by using a Qubit 2.0 Fluorometer (Invitrogen, Carlsbad, CA) as well as by quantitative PCR (KAPA Biosystems, Wilmington, MA, USA).

The sequencing libraries were clustered on 1 flowcell lane. After clustering, the flowcell was loaded on the Illumina HiSeq instrument (4000 or equivalent) according to manufacturer’s instructions. The samples were sequenced using a 2x150bp Paired End configuration. Image analysis and base calling were conducted by the HiSeq Control Software. Raw sequence data (.bcl files) generated from Illumina HiSeq was converted into fastq files and de-multiplexed using Illumina’s bcl2fastq 2.17 software. One mismatch was allowed for index sequence identification.

### Data processing and visualization

Sequence reads were trimmed to remove possible adapter sequences and nucleotides with poor quality using Trimmomatic v.0.36. The trimmed reads were mapped to the Mus musculus GRCm38 reference genome available on ENSEMBL using the STAR aligner v.2.5.2b. The STAR aligner is a splice aligner that detects splice junctions and incorporates them to help align the entire read sequences. BAM files were generated as a result of this step. Unique gene hit counts were calculated by using feature Counts from the Subread package v.1.5.2. The hit counts were summarized and reported using the gene-id feature in the annotation file. Only unique reads that fell within exon regions were counted. If a strand-specific library preparation was performed, the reads were strand-specifically counted. After extraction of gene hit counts, the gene hit counts table was used for downstream differential expression analysis. Using DESeq2, a comparison of gene expression between the control and HCW9302 groups was performed. The Wald test was used to generate p-values and log2 fold changes. Genes with an adjusted p-value < 0.05 and absolute log2 fold change > 1 were called as differentially expressed genes for each comparison.

A gene ontology analysis was performed on the statistically significant set of genes by implementing the software GeneSCF v.1.1-p2. The mgi GO list was used to cluster the set of genes based on their biological processes and determine their statistical significance.

To estimate the expression levels of alternatively spliced transcripts, the splice variant hit counts were extracted from the RNA-seq reads mapped to the genome. Differentially spliced genes were identified for groups with more than one sample by testing for significant differences in read counts on exons (and junctions) of the genes using DEXSeq. For groups with only one sample, the exon hit count tables were provided. The read counts data were analyzed with GraphPad Prism 9. The RAN-seq data can be accessed at: https://doi.org/10.6084/m9.figshare.21718013.v1

### Statistics

Statistical analyses were performed using GraphPad Prism 9. All numerical values are presented as mean values ± SEM. Statistical significance between groups was determined by 1-way ANOVA with Tukey’s correction or by unpaired 2-tailed t test. For each test, a *P* value of less than 0.05 was considered statistically significant.

### Study approval

All animal studies were approved by the Institutional Animal Care and Use Committee of HCW Biologics, Inc.

## Results

### Construction and purification of HCW9302

We used a previously described TF-based protein expression platform (27–29) to create a single-chain polypeptide HCW9302 containing rhIL-2 domains linked to the N- and C-termini of the extracellular domain of human TF (**Figure 1A**). HCW9302 was produced and purified from the culture supernatant of a recombinant CHO-K1 cell line carrying the encoding sequence of the fusion gene by immunoaffinity chromatography as previously described (28). Following deglycosylation of purified HCW9302, a ~56 kDa protein band was observed on SDS-PAGE as expected based on the amino acid sequence of HCW9302 (**Figure 1B**, lane 4), whereas a ≥4 kDa increase in molecular mass was seen in the native glycosylated forms of the protein (**Figure 1B**, lanes 2-3). The chromatograms of HPLC size exclusion chromatography (SEC) are shown in **Figure 1C** for HCW9302 suggesting that the purified HCW9302 was a homodimer.

### Enhanced binding affinity of HCW9302 to IL-2Rα.

**Figure 2:**
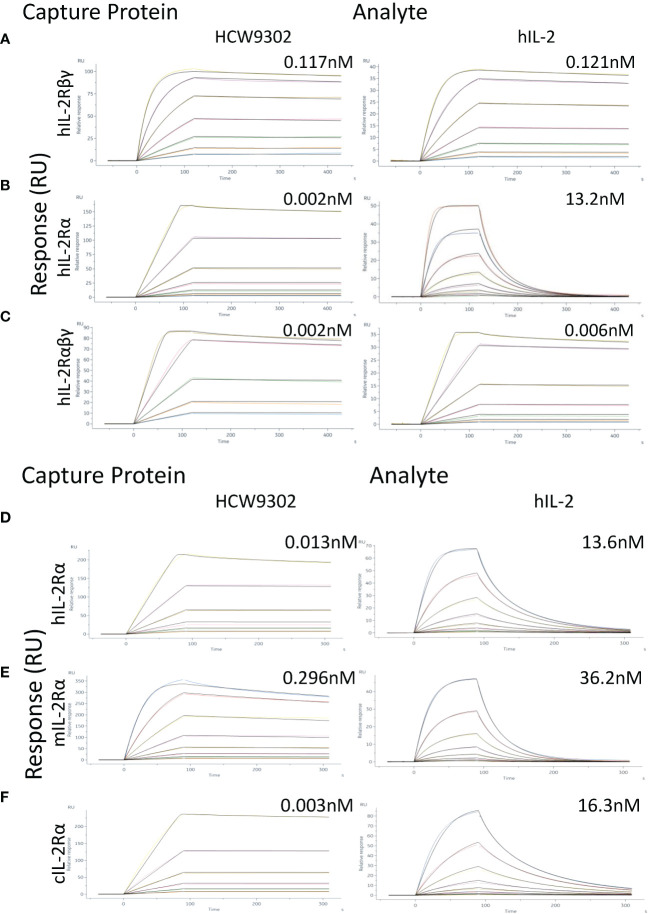
Enhanced binding affinity of HCW9302 to IL-2Rα. **(A)** Fc-tagged human IL-2Rβγ, **(B)** Fc-tagged human IL-2Rα, or **(C)** Fc-tagged human IL-2Rαβγ was captured with anti-human IgG (Fc) antibody on CM5 chips. Binding of different concentrations of HCW9302 or human IL-2 was measured by SPR. **(D)** His-tagged human IL-2Rα, **(E)** His-tagged mouse IL-2Rα, or **(F)** His-tagged cynomolgus IL-2Rα protein was chemically immobilized by amine coupling on CM5 chips. Binding of different concentrations of HCW9302 or human IL-2 was measured by SPR.

Surface plasmon resonance (SPR) analysis was used to compare HCW9302 and rhIL-2 binding affinity to their receptor components, human (h)IL-2Rβγ, hIL-2Rα, or hIL-2Rαβγ. Immobilized hIL-2Rβγ bound to HCW9302 and rhIL-2 with dissociation constants of 117 pM and 121 pM, respectively (**Figure 2A** and **Table 1**). Immobilized hIL-2Ra bound to HCW9302 and rhIL-2 with dissociation constants of 1.99 pM and 13.2 nM, respectively, indicating a >1000-fold increase of binding affinity of HCW9302 compared to rhIL-2 (**Figure 2B** and **Table 1**). Additionally, immobilized hIL-2Rαβγ was found to bind HCW9302 and rhIL-2 with dissociation constants of 2.01 pM and 5.87 pM, respectively, indicating a >2-fold increase of binding affinity of HCW9302 compared to rhIL-2 (**Figure 2C** and **Table 1** **).** These results suggest that HCW9302 has a much higher binding affinity than IL-2 to IL-2Rα whereas similar binding to IL-2Rβγ was observed. The binding affinities to chemically immobilized human, mouse, or cynomolgus monkey IL-2Rα by HCW9302 and rhIL-2 were further confirmed with dissociation constants of 13.2 pM and 13.6 nM, 296 pM and 36.2 nM, 3.19 pM, and 16.3 nM, respectively (**Figures 2D–F** and **Table 2**). Since Tregs constitutively express IL-2Rαβγ and other inactivated immune cells mainly express IL-2Rβγ, these results suggest that low concentrations of HCW9302 may be used to preferentially stimulate and expand Tregs.

**Table 1:** Enhanced binding affinity of HCW9302 to IL-2Rα.

Analyte	IL-2R	ka (1/Ms)	kd (1/s)	KD apparent (pM)
HCW9302	Human IL-2Rβγ	1.45 x 10^6^	170 x 10^-6^	117.0
Human IL-2	Human IL-2Rβγ	1.68 x 10^6^	204 x 10^-6^	121.0
HCW9302	Human IL-2Rα	54000 x 10^6^	107000 x 10^-6^	1.99
Human IL-2	Human IL-2Rα	15 x 10^6^	19700 x 10^-6^	13200.0
HCW9302	Human IL-2Rαβγ	383 x 10^6^	771 x 10^-6^	2.01
Human IL-2	Human IL-2Rαβγ	746 x 10^6^	4380 x 10^-6^	5.87

Fc-tagged human IL-2Rβγ, human IL-2Rα, or human IL-2Rαβγ protein, was immobilized with anti-human IgG (Fc) antibody on CM5 chips, binding affinity of HCW9302 or human IL-2 was measured by SPR.

**Table 2:** Enhanced binding affinity of HCW9302 to IL-2Rα.

Analyte	IL-2R	ka (1/Ms)	kd (1/s)	K_D_ apparent (pM)
HCW9302	Human IL-2Rα	218 x 10^6^	2890 x 10^-6^	13.20
Human IL-2	Human IL-2Rα	17.5 x 10^6^	23900 x 10^-6^	13600.00
HCW9302	Mouse IL-2Rα	3.1 x 10^6^	918 x 10^-6^	296.00
Human IL-2	Mouse IL-2Rα	3.67 x 10^6^	13000 x 10^-6^	36200.00
HCW9302	Cynomolgus IL-2Rα	143 x 10^6^	457 x 10^-6^	3.19
Human IL-2	Cynomolgus IL-2Rα	130 x 10^6^	212000 x 10^-6^	16300.00

His-tagged human, mouse, or cynomolgus IL-2Rα protein, was chemically immobilized by amine coupling on CM5 chips, binding different concentrations of HCW9302 or human IL-2 was measured by SPR.

### Preferential activity of HCW9302 on IL-2Rαβγ bearing cells

**Figure 3:**
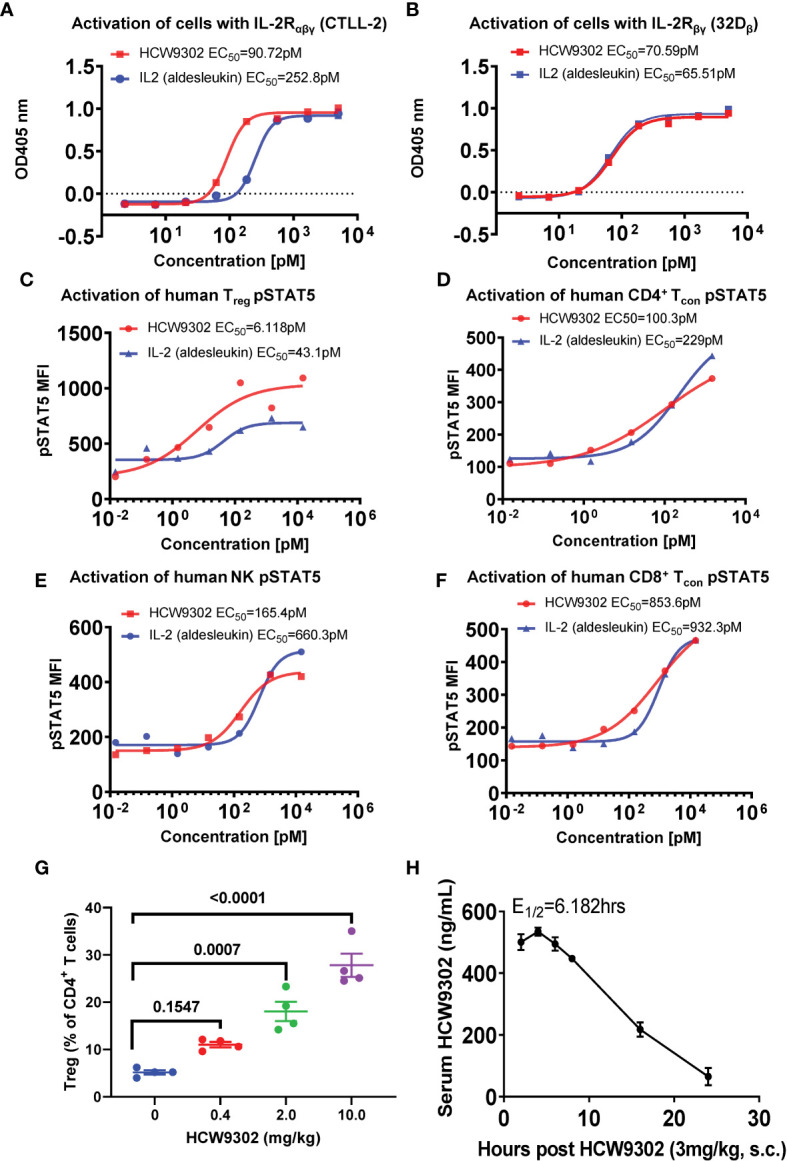
Preferential activity of HCW9302 on IL-2Rαβγ-bearing cells. The biological activities of HCW9302 and rhIL-2 (aldesleukin) were examined with cell proliferation assays using: **(A)** CTLL-2 cells bearing mouse IL-2Rαβγ and **(B)** 32Dβ cells bearing human IL-2Rβ/mouse IL-2Rγ. The ability of HCW9302 and rhIL-2 (aldesleukin) to induce phosphorylation of STAT5 in human lymphocyte subsets: **(C)** Treg, **(D)** CD4^+^ Tcon, **(E)** NK, **(F)** CD8^+^ Tcon cells. **(G)** Expansion of Tregs was induced by HCW9302 in a dose-responsive manner in C57BL/6J mice. **(H)** The predicted fit and actual data for serum HCW9302 levels following the single subcutaneous injections of 3 mg/kg to C57BL/6J mice. Data in **(A–F)** are one represented example from 6 independent experiments. Data in **(G, H)** are expressed as mean ± SEM (n = 3-4). Statistical analysis in **(G)** was performed using ordinary one-way ANOVA with Tukey’s multiple comparisons test.

The IL-2 biological activities of HCW9302 and aldesleukin, clinical grade rhIL-2, were evaluated with cell proliferation assays using cell lines expressing mouse IL-2Rαβγ (CTLL-2) or human IL-2Rβ/mouse IL-2Rγ (32Dβ) (31). As shown in **Figures 3A, B**, the ability of HCW9302 to support growth of CTLL-2 and 32Dβ cells was concentration dependent with half-maximal stimulation (EC_50_) of 90.7 pM and 70.6 pM, respectively, compared with 252.8 pM and 65.5 pM, respectively, for aldesleukin. The observed >two-fold difference in relative activity for the IL-2Rαβγ-bearing cell line but no difference in relative activity for the IL-2Rβγ-bearing cell line comparing HCW9302 and aldesleukin was consistent with the SPR binding affinity observed with purified IL-2R complexes (**Figure 2** and **Tables 1**, **2**).

We further compared the *in vitro* activities of HCW9302 and aldesleukin by inducing downstream IL-2 receptor signaling in human lymphocyte subsets. As shown in **Figures 3C–F**, the ability of HCW9302 to induce phosphorylation of STAT5 in human Treg, CD4^+^ conventional T (Tcon), NK, and CD8^+^ Tcon cells was concentration dependent with half-maximal stimulation (EC_50_) of 6.12, 100.3, 165.4, and 853.6 pM, respectively, compared to aldesleukin with EC_50_ values of 43.1, 229, 660.3, and 932.3 pM, respectively. Thus, compared to rhIL-2, HCW9302 exhibited a >7-fold difference in activating Tregs (**Figure 3C**) and a >4-fold difference in activating NK cells (**Figure 3E**). In contrast, there were <2.5-fold differences in the abilities of HCW9302 and rhIL-2 to activate CD4^+^ Tcon, or CD8^+^ Tcon cells (**Figures 3D, F**). Collectively, these results suggested that HCW9302 exhibited more potent activity in stimulating IL-2Rαβγ-expressing Tregs compared to rhIL-2.

### Pharmacodynamic (PD) and pharmacokinetic (PK) profiles of HCW9302 *in vivo*

Since IL-2 has a short serum half-life (<15 min) (35, 36), we wanted to investigate whether HCW9302 has improved biologic activity *in vivo*. PD and PK parameters of HCW9302 were determined in C57BL/6J mice after subcutaneous injection. Female mice were first injected subcutaneously with 0, 0.4, 2, or 10 mg/kg HCW9302 and splenocytes were analyzed with flow cytometry 4 days after treatment. The percentage of CD4^+^CD25^+^Foxp3^+^ Tregs among the CD4^+^ T-cell population was increased in a dose-dependent manner with HCW9302 and, in the 2 mg/kg and 10 mg/kg treatment groups, were significantly higher than that of the PBS control (**Figure 3G**). No overt adverse effects were observed in this HCW9203 dose range.

To assess HCW9302 PK parameters, female C57BL/6J mice were injected subcutaneously with 3 mg/kg of HCW9302, and blood was collected at various time points from 2 to 24 h post injection. Serum concentrations of HCW9302 were evaluated using ELISA formats with an anti-human tissue factor antibody for capture and an anti-human IL-2 antibody for detection. The predicted fit and actual data for serum HCW9302 concentrations following a single 3 mg/kg subcutaneous injection in C57BL/6 mice are shown in **Figure 3H**. Pharmacokinetic analysis showed a maximal serum concentration of 0.535 μg/mL four hours after dosing and a terminal half-life of 6.2 h (**Table 3**). The apparent clearance (Cl/F) of HCW9302 was 380.6 mL/h/kg and the apparent volume of distribution (Vz/F) was 3395 mL/kg (**Table 3**). These results demonstrate that HCW9302 exhibited a longer serum half-life with more favorable pharmacokinetic properties than rhIL-2.

**Table 3:** Improved pharmacokinetic profile of HCW9302 in mice.

Term half-life	C_max_	T_max_	AUC_last_	AUC_INF_	Cl/F	Vz/F
(h)	(µg/mL)	(h)	(h*µg/mL)	(h*µg/mL)	(mL/h/kg)	(mL/kg)
6.182	0.5348	4	7.2989	7.8822	380.602	3395.4

### HCW9302 activates and expands Treg cells

**Figure 4:**
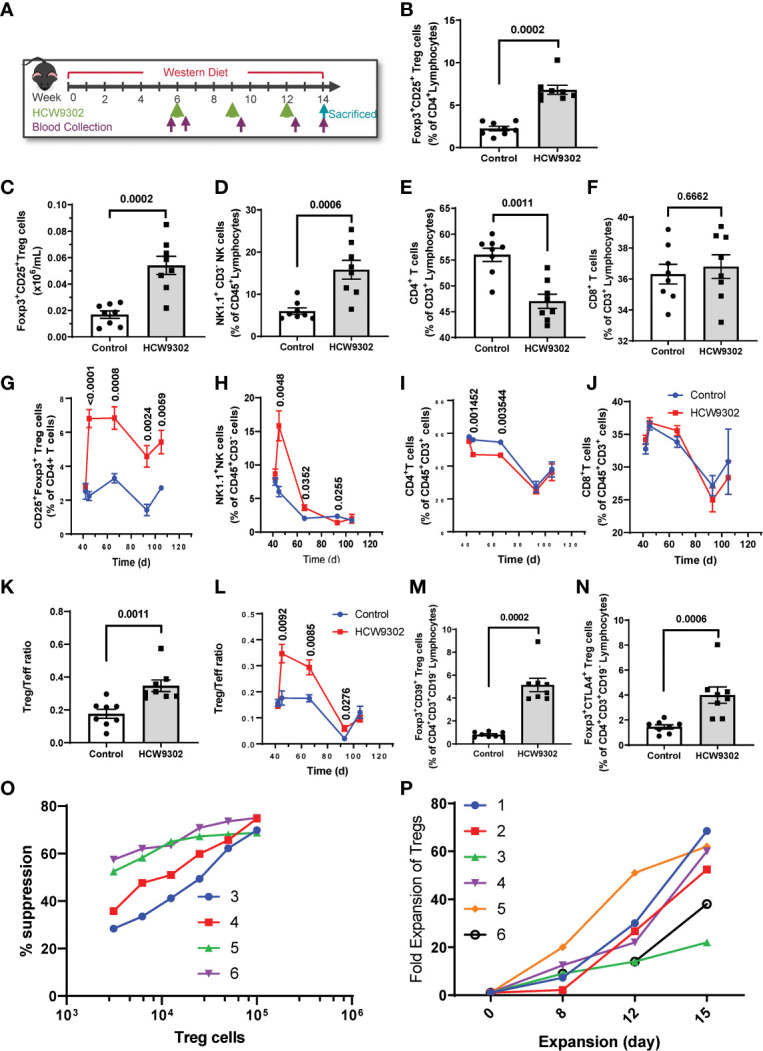
Activation and expansion of Treg cells in HCW9302-treated *ApoE* deficient mice. **(A)** Schematic study design in *ApoE* deficient mice. The mouse blood lymphocyte subsets: **(B)** CD4^+^CD25^+^Foxp3^+^ Treg cells, **(C)** Absolute numbers of Treg cells, **(D)** NK1.1^+^CD3^-^ NK cells, **(E)** CD4^+^ T cells, **(F)** CD8^+^ T cells from 3 days post 1st dose were analyzed by flow cytometry. A dynamic analysis of mouse blood lymphocyte subsets: **(G)** CD4^+^CD25^+^Foxp3^+^ Treg cells, **(H)** NK1.1^+^CD3^-^ NK cells, **(I)** CD4^+^ T cells, **(J)** CD8^+^ T cells, from pre-dose to the end of study was compared between control and HCW9302 treated groups. The mouse CD4^+^Foxp3^+^ Treg to CD4^+^Foxp3^-^ Teff ratio: **(K)** from 3 days post 1st dose, **(L)** a dynamic analysis, was compared between control and HCW9302 treated groups. The mouse suppressive marker positive: **(M)** CD4^+^CD39^+^Foxp3^+^ and **(N)** CD4^+^CTLA4^+^Foxp3^+^, Treg subsets were compared between control and HCW9302 treated groups. **(O)** Ex vivo expansion of human CD4^+^CD25^+^CD127^low^ Tregs from 6 healthy donors with HCW9302. **(P)** Suppression of proliferation of effector CD4^+^CD25^-^ T cells (Tresp) from 4 healthy donors with ex vivo-expanded human CD4^+^CD25^+^CD127^low^ Tregs from the same donors by HCW9302. Data are expressed as mean ± SEM (n = 7-9). Statistical analysis in **(B–F, K)**, and **(M, N)**, using a 2-tailed, unpaired t test, in **(G–J, L)**, using multiple unpaired t tests.

We then evaluated the ability of HCW9302 to affect Treg responses in an inflammatory disease-relevant mouse model. Six-week-old *ApoE* deficient mice were fed with a high-fat Western diet (WD) for 6 weeks to induce aortic atherosclerosis. HCW9302 was injected subcutaneously at 3 mg/kg once every 3 weeks for three doses while the mice continued a Western diet (**Figure 4A**). PBS-treated mice served as controls. Blood samples were collected from the submandibular vein one day before the dosing, three days post each injection, and 2 weeks post the 3^rd^ injection (**Figure 4A**), and lymphocyte subsets were analyzed by flow cytometry. The results from 3 days post 1^st^ dose are shown in **Figures 4B–F** (and **Supplementary Figures 1A–C**). The percentages of blood Treg and NK1.1^+^CD3^-^ NK cells were significantly increased by HCW9302 treatment (**Figures 4B, D** and **Supplementary Figures 1A, B**). The absolute numbers of Treg cells were significantly increased by HCW9302 treatment (**Figure 4C**). In contrast, HCW9302 treatment reduced the percentage of blood CD4^+^ T cells (**Figure 4E**) and had no effect on blood CD8^+^ T cell percentages (**Figure 4F**). Dynamic analysis of blood lymphocyte subset changes from pre-dose to the end of study is shown in **Figures 4G–J**. The percentage of CD4^+^CD25^+^Foxp3^+^ Tregs increased during the entire dosing period by HCW9302 treatment (**Figure 4G**). The percentage of NK1.1^+^CD3^-^ NK cells also increased after initial HCW9302 treatment but returned to control levels by the end of study (**Figure 4H**). The percentage of CD4^+^ T cells were reduced after initial two doses of HCW9302 treatment but returned to control levels after the 3^rd^ dose (**Figure 4I**), whereas treatment did not affect the percentage of blood CD8^+^ T cells (**Figure 4J**). In addition, the ratio of blood CD4^+^Foxp3^+^ Treg to CD4^+^Foxp3^-^ effector T (Teff) cells was found to increase during the HCW9302 dosing period in *ApoE* deficient mice (**Figures 4K, L** and **Supplementary Figure 1D**). HCW9302 treatment also increased CD39^+^ and CTLA4^+^ Treg subsets (**Figures 4M, N** and **Supplementary Figures 1E, F**), suggesting suppressive functions of Tregs were enhanced by HCW9302.

To further evaluate whether HCW9302 can activate and expand human Tregs, Tregs were isolated from healthy human donor PBMCs and treated ex vivo with anti-CD3/CD28 beads, rapamycin, and HCW9302. The human CD4^+^CD25^+^CD127^low^ Tregs were expanded over 60-fold after 15 days in culture (**Figure 4O**). The suppressive ability of human Tregs was measured as a decrease of proliferation of effector CD4^+^CD25^-^ T cells (T responder cells, Tresp) in the presence of different concentrations of HCW9302-expanded Tregs (Treg : Tresp ratios of 1:32, 1:16, 1:8, 1:4, 1:2, and 1:1). As shown in **Figure 4P**, the ex vivo HCW9302-expanded human Tregs potently suppressed Tresp from different healthy donors. Collectively, this data demonstrates that HCW9302 is capable of activating and expanding Tregs in metabolically dysfunctional mice *in vivo* and human Tregs from peripheral blood *in vitro*.

### Attenuation of progression of atherosclerosis by HCW9302 in mice

**Figure 5:**
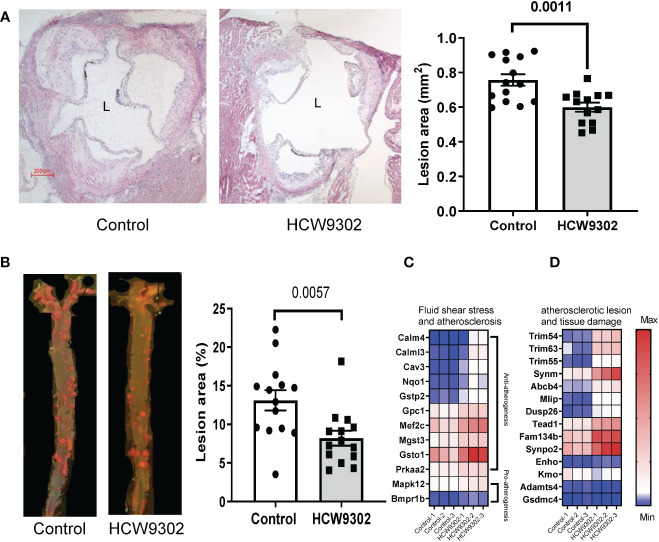
Attenuation of progression of atherosclerosis. **(A)** Histochemical staining of aorta root sections and graphical comparisons of the atherosclerotic lesion formation (H&E) between control and HCW9302 treated *ApoE* deficient mice fed with a Western diet (WD). **(B)** En face analysis of atherosclerotic lesions in the aorta including the arch, thorax, and abdomen from control and HCW9302 treated *ApoE* deficient mice fed with a Western diet (WD). **(C)** Heatmap analysis of HCW9302-mediated increase of anti-atherosclerotic transcriptomes and **(D)** reduction of pro-atherosclerotic transcriptomes in aortas of *ApoE*-deficient mice. L=lumen. Data in **(A, B)** are expressed as mean ± SEM (n = 13-14). Statistical analysis using a 2-tailed, unpaired t test. In **(C, D)**, the Wald test was used to generate p-values and log2 fold changes for RNA seq. Genes with an adjusted p-value < 0.05 and absolute log2 fold change > 1 were called as differentially expressed genes for each comparison.

To determine whether HCW9302 can alleviate the progression of atherosclerosis, 6-week-old *ApoE* deficient mice were fed with WD for 6 weeks after which they were treated with three doses of HCW9302 or PBS control (once every 3 weeks). During the treatment period, the mice were continuously on WD (**Figure 4A**). No adverse effects were observed in either group throughout the study. There were no significant differences in body weights, plasma triglycerides, and plasma LDL, between control and HCW9302 treated groups throughout the study (**Supplementary Figures 2A–C**). By the end of the study, aortic sections from *ApoE* deficient mice, were examined to observe that HCW9302 caused a significant reduction in atherosclerotic lesion formation in the aortic sinus compared with control mice (**Figure 5A**). The aortas including parts of the arch, thorax, and abdomen were collected for en-face analysis. A significant reduction in aortic plaque burden in HCW9302-treated mice was observed when compared with control mice (**Figure 5B**). Similar results were observed in *Ldlr* deficient mice fed WD where HCW9302 treatment markedly attenuated further progression of atherosclerosis (data not shown).

In RNA-seq analysis performed on whole aorta of WD-fed *ApoE* deficient mice, we found that HCW9302 treatment resulted in changes of gene expression profiles consistent with reduced atherosclerotic lesions and enhanced tissue regeneration. Twelve transcripts associated with fluid shear stress and atherosclerosis based on KEGG (KEGG pathway map 05418) were increased. Ten of these genes are related to anti-atherogenesis (*Calm4, Calml3, Cav3, Nqo1, Gstp2, Gpc1, Mef2c, Mgst3, Gsto1*, and *Prkaa2*) and two of the genes are related to pro-atherogenesis (*Mapk12* and *Bmpr1b*) (**Figure 5C**). The expression of eleven genes (*Trim54, Trim63, Trim55*, *Synm, Abcb4, Mlip, Dusp26, Tead1, Fam134b, Synpo2*, and *Enho*) related to reduction of atherosclerotic lesions and tissue damage (37–44) were increased (**Figure 5D**). Three transcripts (*Kmo, Adamts4*, and *Gsdmc4*) related to the increase of atherosclerotic lesions and tissue damage (45–49) were reduced (**Figure 5D**).

### Reduction of inflammation in aorta through upregulation of Tregs, M2 macrophages, and MDSCs

**Figure 6:**
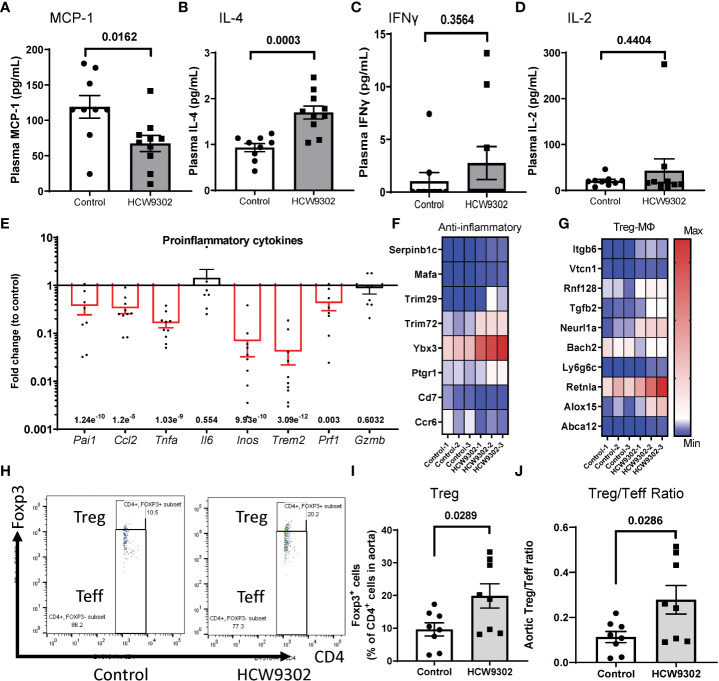
Induction of anti-inflammation phenotypes and reduction of pro-inflammation phenotypes in blood and aorta following HCW9302 treatment. Reduction of blood inflammatory cytokine **(A)** MCP-1 and **(B)** increase of Th2 cytokine IL-4 but no effects on Th1cytokines: **(C)** INFγ, **(D)** IL-2, following HCW9302 treatment relative to control was analyzed with ELISA. **(E)** Reduction of pro-inflammatory cytokine gene expression in aortas following HCW9302 treatment relative to control was analyzed by q-PCR. **(F)** HCW9302-mediated increases in anti-inflammation transcriptomes and reduction of pro-inflammation transcriptomes. **(G)** Increased Treg cell activation and expansion, MDSCs/M2 macrophage polarization, macrophage efferocytosis, and macrophage cholesterol efflux transcriptomes, and reduction of Treg cell suppressive transcriptomes in aortas following HCW9302 treatment. **(H)** The representative flow cytometry plots of mouse aortic CD4^+^Foxp3^+^ Treg cells and CD4^+^Foxp3^-^ Teff cells. The mouse aortic lymphocyte subsets: **(I)** CD4^+^Foxp3^+^ Treg cells, **(J)** CD4^+^Foxp3^+^ Treg and the CD4^+^Foxp3^-^ Teff ratio, were compared between control and HCW9302-treated *ApoE* deficient mice fed with WD. Data are expressed as mean ± SEM (n = 8-11). Statistical analysis in **(A–F)**, **(I, J)** was performed using a 2-tailed, unpaired t test. In **(F, G)**, the Wald test was used to generate p-values and log2 fold changes for RNA seq. Genes with an adjusted p-value < 0.05 and absolute log2 fold change > 1 were called as differentially expressed genes for each comparison.

Inflammation plays a major role in progression of atherosclerosis (3). In the WD-fed *ApoE^-/-^* mice treated with HCW9302, we found that blood inflammatory cytokine MCP-1 was reduced and Th2 cytokine IL-4 was increased whereas Th1 cytokines IFNγ and IL-2 (**Figures 6A–D**), as well as other inflammation cytokines: IL-1β, GM-CSF, IL-6, IL-10, TNFα, and IL-12p70 (**Supplementary Figures 2D–I**) were not changed by ELISA. Locally, HCW9302 was able to reduce the expression of genes associated with inflammation in the aortas of WD-fed *ApoE* deficient mice. Results from q-PCR showed that the expression of the inflammatory genes *Pai1, Ccl2, Tnfα, Inos1, Trem2*, and *Prf1* (but not *IL6* and *Gzmb*) were reduced (**Figure 6E**). RNA-seq analysis of whole aorta RNA showed that the expression of six genes (*Serpinb1c, Mafa*, *Trim29, Trim72/MG53, Ybx3*, and *Ptgr1*) related to anti-inflammation responses (50–54) were elevated and the expression of two genes (*Ccr6* and *CD7*) related to inflammation (55, 56) were reduced with HCW9302 treatment (**Figure 6F**).

Additionally, RNA-seq of whole aorta demonstrated upregulated expression of Treg cell-, M2 macrophage-, and MDSC-associated genes in the aorta after HCW9302 treatment. Expression of five genes (*Itgb6*, *Vtcn1, Rnf128/Grail, Tgfb2*, and *Neurl1a*) related to Treg cell activation and expansion (57–62) were elevated and one gene (*Bach2*) related to inhibition of Treg cell activation (63–65) was reduced (**Figure 6G**). In contrast, the expression of four genes (*Ly6g6c*, *Retnla/Fizz1, Alox15*, and *Abca12*) associated with MDSCs (66, 67), M2 macrophage polarization (68), macrophage efferocytosis (69, 70), and macrophage cholesterol efflux (71) were upregulated (**Figure 6G** **)**.

**Figure 7:**
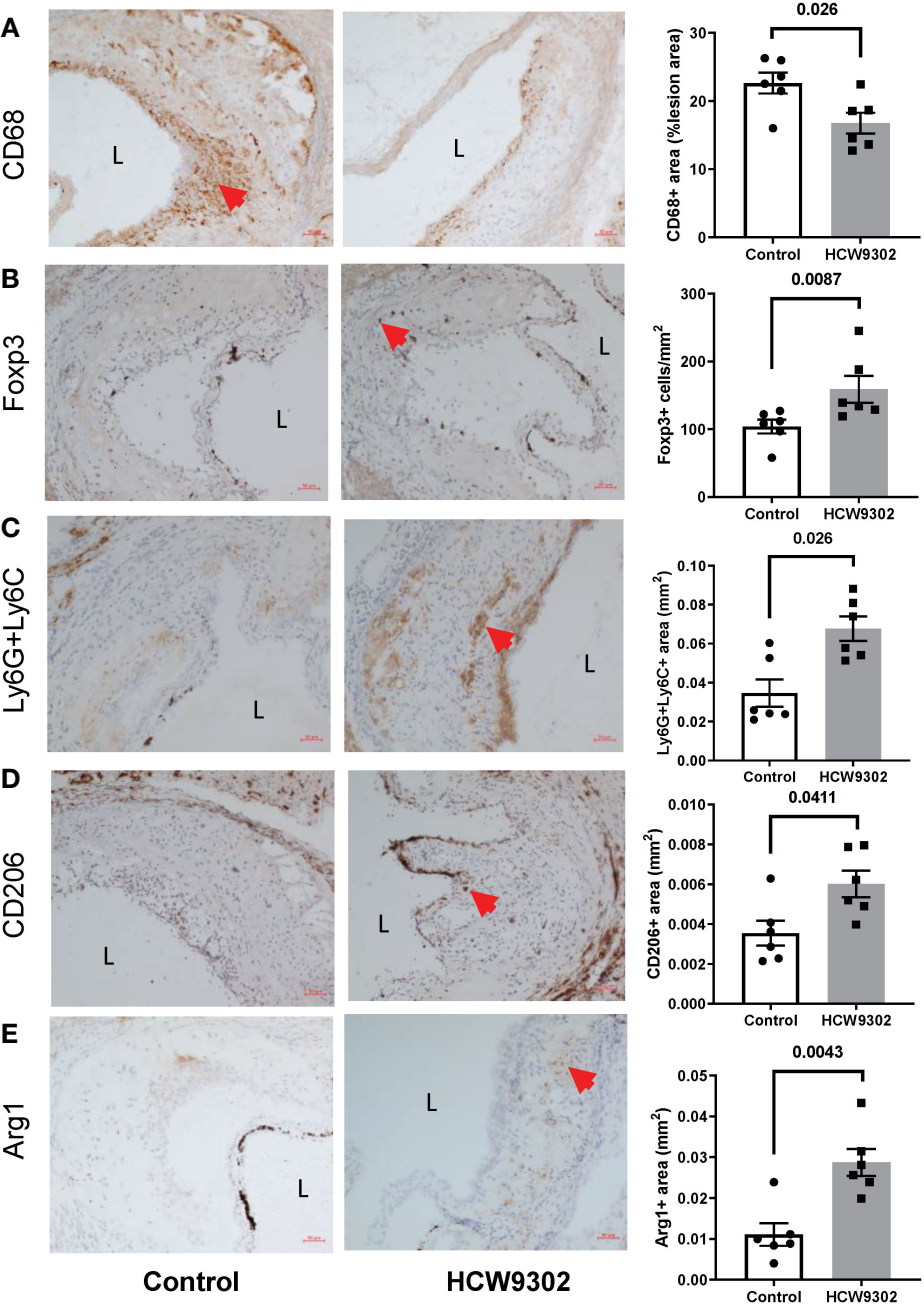
Immunohistochemical evaluation of Treg cells and macrophages in aortic root of HCW9302-treated *ApoE* deficient mice. Mouse aortic macrophages and Treg cells: **(A)** CD68^+^ macrophages, **(B)** Foxp3^+^ Treg cells, **(C)** Ly6g6c^+^ MDSCs, **(D)** CD206^+^ M2 macrophages, and **(E)** Arg1^+^ macrophages/MDSCs were compared between control and HCW9302 treated *ApoE* deficient mice fed with WD. L=lumen; arrow bar indicated the positive staining cells. Data are expressed as mean ± SEM (n = 6). Statistical analysis in **(A–E)** was performed using a 2-tailed, unpaired t test.

Lymphocytes from whole aorta (arch, ascending and descending) of WD-fed *ApoE* deficient mice were also isolated for flow cytometry analysis. The percentage of CD4^+^Foxp3^+^ Treg cells and the Treg/Teff ratio were increased by HCW9302 treatment (**Figures 6H–J**). The percentages of other CD4^+^ T subsets including Th, Th1, Th2, and Th17 cells were unchanged (**Supplementary Figures 3A–H**). The percentages of CD8^+^ T cells, NK cells, and CD11b^+^F4/80^+^ macrophages were unchanged (**Supplementary Figures 4A–F**). Further immunohistochemical staining of aortas from WD-fed *ApoE* deficient mice revealed that the number of CD68^+^ macrophages were reduced (**Figure 7A**), but the number of Foxp3^+^ Tregs, Ly6G^+^Ly6C^+^ MDSCs, and CD206^+^ M2 macrophages were increased in the intima of the mouse aortic sinus following treatment with HCW9302 (**Figures 7B–D**). Arginase 1 which is a key effector and biomarker of both M2 macrophages and MDSCs was significantly increased in aorta by HCW9302 treatment (**Figure 7E**).

## Discussion

Atherosclerosis is a chronic inflammatory disease and both the innate and adaptive immune systems have been shown to play a role in either accelerating or curbing this disease (72). CD8^+^ T cells, Th1 cells, type 1 innate lymphoid cells (ILC1), and M1 macrophages have been implicated as pro-atherogenic (72–76), whereas Tregs, type 1 regulatory T cells (Tr1), M2 macrophages, MDSCs, and ILC2 have been demonstrated to be antiatherogenic (66, 67, 75–77). In contrast, NK cells were shown to have no direct effect on the natural development of hypercholesterolemia-induced atherosclerosis (78). Interestingly, Tregs modulate numerous macrophage activities in atherosclerosis. Tregs suppress M1 macrophages, polarize M1 to M2 macrophages (79), and promote macrophage efferocytosis during inflammation resolution (15), thereby enhancing the pro-resolving capacity of macrophages in plaques (16), and facilitating cross-talk with MDSCs (80). Together Tregs, M2 macrophages, and MDSCs attenuate atherosclerosis by reducing inflammation, atherosclerotic lesions, and tissue damage (6, 67, 81). Thus, there is significant interest in expanding Tregs as a therapeutic approach to treat atherosclerosis. Since IL-2, a common gamma chain cytokine, plays a pivotal role in Treg development and expansion, multiple IL-2-based therapies to expand Tregs cells and minimize IL-2 immune stimulatory activities on effector cells are being developed (20). These include the use of low-dose IL-2, antibody-cytokine conjugates, IL-2 muteins, and IL-2 fusions to large carrier molecules such as immunoglobulin Fc, albumin or polyethylene glycol (17). In this study, we show that HCW9302, a single-chain IL-2/TF/IL-2 fusion created by our platform TF scaffold protein technology (27–29), had a high affinity to human, cynomolgus, and mouse CD25 compared to rhIL-2, rhIL-2 muteins, genetically fused rhIL-2/Fc, or large carrier molecules-coupled rhIL-2 (82, 83). HCW9302 also exhibits a long serum half-life compared with rhIL-2. These unique properties may contribute to the ability of subcutaneously administered HCW9302 to preferentially activate and expand Tregs in mice in a well-tolerated dose range without activating proatherogenic CD4^+^ T cells (84). This result also demonstrates that activation of Tregs through enhanced binding of CD25 may have advantages over “mutein” approaches to block or reduce IL-2 binding to IL-2Rβγ on effector cells (20). Our results are consistent with those of previous studies that used modified IL-2 to induce Tregs and to polarize M2 macrophages for anti-atherogenic effects in *ApoE* deficient mice (21, 85). In addition to the activation and expansion of Tregs, we found HCW9302 also expanded the percentage of NK cells and reduced levels of CD4^+^ T cells in the blood and expanded M2 macrophage and MDSC levels in the atherosclerosis plaques of WD-fed *ApoE* deficient mice. Additional studies are underway to further investigate the interplay between treatment-mediated Treg activation, M2 macrophage polarization and MDSC induction in the atherosclerotic plaques on the anti-atherosclerotic activity of HCW9302.

In addition to their anti-inflammatory function, Tregs are involved in supporting tissue homeostasis and repair (86). It is conceivable that HCW9302-mediated expansion of Tregs also enhances the tissue-repair capacity to reduce atherosclerotic lesions. This is consistent with our observations in this study that HCW9302 treatment up-regulated gene transcripts related to reduction of atherosclerotic lesions and tissue damage as well as those related to reduction of fluid shear stress and atherosclerosis. Thus, HCW9302 treatment or *in vivo* expansion of Tregs by HCW9302 could be an effective approach to reduce inflammation and treat autoimmune diseases. Currently, cGMP HCW9302 clinical material is available, and we are at the late IND-enabling stages to enter clinical development of HCW9302 for an autoimmune disease.

## Data availability statement

The RNA-seq data presented in the study are deposited in the SRA repository with accession number PRJNA925136.

## Ethics statement

The animal study was reviewed and approved by Institutional Animal Care and Use Committee of HCW Biologics Inc.

## Author contributions

XZ, QL, VG, CS, and BL performed *in vivo* and *in vitro* experiments. CG and NS performed *in vitro* experiments. ZW participated in histology and IHC data analysis. XZ, LK, LY, CE, GM, and JE participated in HCW9302 production. PC, VG, and PR participated in revision of the manuscript. XZ and HW were responsible for HCW9302 design, study design, conceptualization, data collection, analysis, interpretation, supervised the study and wrote the manuscript. All authors contributed to the article and approved the submitted version.
