# Supplementary material for: A novel interleukin-2-based fusion molecule, HCW9302, differentially promotes regulatory T cell expansion to treat atherosclerosis in mice
Source: Front Immunol. 2023 Jan 25;14:1114802. doi: 10.3389/fimmu.2023.1114802 (PMC9907325; doi:10.3389/fimmu.2023.1114802)
Supplement: Supplementary file 1 [file DataSheet_1.docx]

***Supplementary Material***

**A novel IL-2-based fusion molecule, HCW9302, differentially promotes Treg expansion to treat atherosclerosis in mice**

**Xiaoyun Zhu,^1†^ Qiongzhen Li,^1†^ Varghese George,^1†^ Catherine Spanoudis,^1^ Crystal Gilkes,^1^ Niraj Shrestha,^1^ Bai Liu,^1^ Lin Kong,^1^ Lijing You,^1^ Christian Echeverri,^1^ Liying Li,^1^ Zheng Wang,^1^ Pallavi Chaturvedi,^1^ Gabriela Muniz,^1^ Jack O. Egan,^1^ Peter R. Rhode^1^ and Hing C. Wong^1*^**

^1^HCW Biologics Inc., Miramar, FL, 33025 USA

^†^These authors contributed equally to this work and share first authorship.

***Correspondence:** Corresponding author: Hing C. Wong, Ph.D., HCW Biologics Inc., 2929 N. Commerce Parkway, Miramar, FL, 33025 USA, E-mail: hingwong@hcwbiologics.com

**Detailed Methods**

*Construction and production of HCW9302 fusion protein.* HCW9302 is a recombinant fusion protein, constructed by fusing one human IL-2 domain to the N-terminus of the soluble extracellular domain of human tissue factor, and another human IL-2 domain to the C-terminus of the human tissue factor domain (Figure 1A). The corresponding coding DNA sequences were synthesized (Genewiz), cloned into pMSGV-1 modified expression vectors (1) and transfected into CHO.K1 cells (ATCC, CCL-61). HCW9302 is expressed in CHO cells and secreted of the fusion protein into the culture media. HCW9302 expression was then detected with product specific ELISA formats using an anti-human tissue factor antibody (HCW Biologics, HCW9101) for capture and an anti-human IL-2 antibody (R&D Systems, BAF202) for detection. Production cell banks for HCW9302 were generated from stably transfected clonal cell lines following limited dilution cloning. Subsequent fusion protein production was conducted using fed-batch methods with chemically defined media in shake flasks or stir tank bioreactors. HCW9302 was purified from clarified culture media using immunoaffinity chromatography with anti-TF Ab-conjugated Sepharose resin, and then buffer-exchanged into PBS. A GMP-suitable manufacturing process (scaled from 2 L to 200 L) was developed for HCW9302 consisting of immunoaffinity chromatography, low pH viral inactivation/depth filtration, multimodal chromatography, nanofiltration, and ultrafiltration/diafiltration steps employing commercially scalable methods. The purified HCW9302 product was characterized and released using qualified test methods per established specifications.

*Surface plasmon resonance.* Binding affinities of HCW9302 and human IL-2 (Acro, S13-H5113) were measured by surface plasmon resonance on human IL-2Rαβγ, IL-2Rβγ, or IL-2Rα on a Biacore 8K (Cytiva). The Fc-tagged human IL-2Rαβγ (Acro, ILG-H5257), IL-2Rβγ (Acro, ILG-H5254), or IL-2Rα (Acro, ILA-H5251) were captured by immobilized anti-human IgG (Fc) antibody (Cytiva, 29234600) on CM5 chips (Cytiva, BR100530). To measure binding to IL-2Rs, 2-fold dilutions of HCW9302 (0.061-0.977 nM) or IL-2 (0.061-3.906 nM) for IL-2Rαβγ binding, of HCW9302 (0.488-31.25 nM) or IL-2 (0.488-31.25 nM) for IL-2Rβγ binding, and of HCW9302 (0.049-3.125 nM) or IL-2 (0.195-25 nM) for IL-2Rα binding, were injected over the chip surface for 120 seconds and dissociations were monitored for 300 seconds. The surface was regenerated after each injection by washing with 3 M magnesium chloride for 30 seconds. The binding curves were fitted using a 1:1 fitting model. To confirm binding affinities of HCW9302 and human IL-2 on IL-2Rα, his-tagged human (Acro, ILA-H5251), mouse (Acro, ILG-M52H9), or cynomolgus (Sino, 90265-C08H) IL-2Rα were chemically immobilized by amine coupling on CM5 chips. Two-fold dilutions of HCW9302 (0.195-6.25 nM) or IL-2 (0.195-25 nM) for human IL-2Rα binding, of HCW9302 (0.195-25 nM) or IL-2 (0.195-25 nM) for mouse IL-2Rα binding, and of HCW9302 (0.195-6.25 nM) or IL-2 (0.195-25 nM) for cynomolgus IL-2Rα binding, were injected respectively over the chip surface for 90 seconds and dissociations were monitored for 210 seconds. The surface was regenerated after each injection by washing with 3 M magnesium chloride for 30 seconds. The binding curves were fitted using a 1:1 fitting model.

*In vitro verification of HCW9302 activity.* CTLL-2 (ATCC, TIB-214) and 32Dβ cells (2) were maintained in IMDM supplemented with 10% FBS and 25 ng/ml of human recombinant IL-2 (Peprotech, 200-02). For cell assays, CTLL-2 and 32Dβ cells were washed 5 times and seeded at 2 x 10^4^ cells/well in 96-well plates in IMDM with 10% FBS. Purified HCW9302 was added to the wells at 1:3 serial dilutions and cells were incubated for 72 hours at 37^o^C in a CO_2_ incubator. PrestoBlue proliferation reagent (ThermoFisher, A13261, 20 µL/well) was added. After 4 hours, absorbance was measured at 570/610 nm to determine cell proliferation based on reduction of the PrestoBlue™ reagent to resorufin by metabolically active cells. The bioactivity of human recombinant IL-2 (Proleukin® (aldesleukin), NDC 65483-116-07, or Peprotech, 200-02) was assessed as a positive control.

Peripheral blood mononuclear cells (PBMC) from heathy donors were isolated from whole blood buffy coats by Ficoll Paque Plus (Millipore/Sigma, GE17144003). PBMCs were treated with ammonium chloride-potassium (ACK) lysing buffer (Thermo Fisher Scientific, A1049201) to remove red blood cells. Cells were washed with IMDM-10% FBS and counted. Cells (1.8 x10^6^ in 100 µl/tube) were seeded to the flow tubes and incubated with 50 μl of l/10-diluted HCW9302 or IL2 (Proleukin® (aldesleukin), NDC 65483-116-07) (15000, 1500, 150, 15, 1.5, 0.15, or 0 pM) and 50 µl of anti-CD8-BV605 (BioLegend, 344742; 1:50). Cells were incubated for 30 min at 37^o^ C. Pre-warmed BD Phosflow Fix Buffer I (200 µl) (BD Biosciences, 557870) was added for 10 min at 37^o^ C to stop the stimulation. Cells (4.5 x10^5^ cells/100 µl) were transferred to V-shape 96-well plates and were spun down followed by permeabilization with 100 µl of pre-cooled BD Phosflow Perm Buffer III (BD Biosciences, 558050) for 30 min in ice. Cells were washed twice with 200 µl of FACS buffer and stained with a panel of fluorescent antibodies: anti-CD25-PE (BD Biosciences, 555432; 1:100), CD4-PerCP-Cy5.5 (BD Biosciences, 560650; 1:50), CD56-BV421 (BioLegend, 362552; 1:100), and pSTAT5a-AF488 (BD Biosciences, 612598; 1:100), to distinguish different lymphocyte subpopulations and pSTAT5a status. Cells were spun down and resuspended in 200 µl of FACS buffer for FACSCelesta analysis.

Human CD4^+^CD127^low^CD25^+^ regulatory T cells (Tregs) and CD4^+^CD25^-^ responder T cells were isolated from fresh PBMCs of healthy donors using EasySep™ Human CD4^+^CD127^low^CD25^+^ Regulatory T Cell Isolation Kit (Stemcell, 18063). Tregs were expanded in RPMI-1640 supplemented with 10% FBS (R10), Dynabeads Human T-activator CD3/CD28 (Thermo Fisher, 11131D) at a bead to total cell ratio of 4:1, 50 nM HCW9302. Cells were counted and stained to check purity at days 1, 5, 10, and 15. Functionality of human Treg product was assessed on the basis of the ability to suppress proliferation of autologous CD4^+^CD25^−^ T effector cells (responder cells, Tresp) as described (3). Briefly, cryo-stored Tresp cells from the same donors were thawed, washed in turn with 10 ml R10 and PBS. Tresp cells were stained with CellTrace Violet cell proliferation kit (Thermo Fisher, C34557) at 1/1000 dilution in 1 ml of PBS. After incubation for 20 min at 37◦ C, 5 ml R10 was added, and cells were incubated for an additional 5 min. The cells were centrifuged, resuspended in R10 and analyzed by flow cytometry to confirm that the cells were labeled. Labeled Tresp cells were washed once in 10 ml of cold R10 and resuspended in prewarmed R10 for plating. Tregs were seeded in a round bottom 96 well cell culture plates to reach Treg:Tresp ratios of 1:32, 1:16, 1:8, 1:4, 1:2, 1:1, and 1:0 in a volume of 100 µl. Labeled Tresp cells (1 × 10^5^) were added to the plate and Dynabeads Human T-activator CD3/CD28 (Thermo Fisher, 11131D) were added at a bead to total cell ratio of 1:75. In order to determine maximum proliferation of Tresp cells, Tresp cells were cultured with or without beads in the absence of Tregs. The final volume was adjusted to 200 µl for all conditions. Plates were incubated at 37◦ C for 5 days. On day 5, the plates were spun down and the individual wells were harvested. Proliferating Tresp cells were defined as the percentage of CellTrace Violet (BV421) ^+^ cells shifted from the original Tresp population. The mean inhibition of proliferation (% suppression) found at the different Treg:Tresp ratios was calculated as (Proliferation_Tresp only_-Proliferation_Tresp with Treg_)/Proliferation_Tresp only_ × 100. A nonlinear fit of the percentage of suppression vs. the number of added Treg for each condition was calculated using GraphPad Prism 9.

*Animals and experimental protocols.* Six-week-old female C57BL/6J (strain# 000664), B6. ApoE deficient mice (B6.129P2-*Apoe^tm1Unc^*/J, strain# 002052), or B6. Ldlr deficient mice (B6.129S7-*Ldlr^tm1Her^*/J, strain# 002207), were purchased from the Jackson Laboratory (Bar Harbor, ME, USA). For atherosclerosis studies, ApoE deficient mice or Ldlr deficient mice were maintained on a high fat Western diet (TD.88137, Envigo), A group of Ldlr-deficient mice was also fed the regular chow diet, 6 weeks later the mice were administered HCW9302 subcutaneously at 3 mg/kg. The mice received 2 additional consecutive doses

of HCW9302 at weeks 9 and 12 while continuing on the Western diet. Control mice received subcutaneous PBS. Mice were euthanized at 20 weeks of age (14 weeks after initiating the Western diet) and aortic atherosclerotic lesions were assessed.

To evaluate the pharmacokinetic profile of HCW9302, female C57BL/6J mice (three mice/time point) were subcutaneously injected with HCW9302 (3 mg/kg) and blood was collected at various time points from 2 to 24 h post injection. Serum concentrations of HCW9302 were evaluated using ELISA formats with an antihuman tissue factor antibody (HCW Biologics, HCW9101) for capture and an anti-human IL-2 antibody (R&D Systems, BAF202) for detection. HCW9302 levels were fit with a one-compartment

model using PK Solutions 2.0 (Summit Research Services, Montrose, CO, USA).

To evaluate the effect of HCW9302 on plasma cytokines, blood was collected through submandibular vein puncture one week after 3^rd^ dose of HCW9302. Plasma was collected and run at a 2-fold dilution with PBS, and analyzed at Eve Technologies (Calgary, Canada) using Mouse Cytokine Proinflammatory Focused 10-Plex Discovery Assay Array (MDF10).

To determinate plasma LDL cholesterol and triglycerides, 12 h fasted blood was collected by submandibular vein puncture. Plasma LDL cholesterol and triglycerides were determined using Crystal Chem mouse LDL cholesterol kit (Cat# 79980, Crystal Chem, Elk Grove Village, IL, USA) and Abcam Triglyceride quantification kit (Cat# ab65336, Cambridge, MA, USA) following manufacturer’s instructions.

*Enface analysis of aortic lesion.* Euthanized ApoE deficient and Ldlr deficient mice were perfused through the left ventricle with 4% paraformaldehyde-sucrose followed by PBS-EDTA. The whole aorta including heart was harvested and fixed in 4% paraformaldehyde-sucrose. The soft/loose perivascular adipose tissue was gently removed from around the aorta from heart to iliac bifurcation. The branching arteries and heart were removed under the dissection microscope at 20-25x magnification using fine iris scissors and delicate forceps. The whole aorta was split longitudinally, pinned in the black wax petri dish, fixed overnight with 4% paraformaldehyde-sucrose at room temperature, rinsed, fixed with 70% ethanol, and stained using Sudan IV staining solution to identify the plaques as described (4). The total aorta surface area and plaque surface area stained with Sudan IV was captured by a dissection microscope (AmScope). Quantification of the Sudan IV-stained lipid rich plaque area was done using Image J software (<https://www.fiji.sc/>).

*Aorta collection and aortic sinus sectioning.* Euthanized ApoE deficient mice were perfused through the left ventricle with cold PBS-EDTA. The whole aorta including heart was harvested. The aorta including parts of arch, thorax, and abdomen were collected for flow cytometric analysis, qPCR, or RNA seq. The heart was cut so that all three aortic valves were in the same geometric plane. The upper portion of the heart was embedded in O.C.T., frozen in the Peltier stage of the cryostat (Leica CM1950 Cryostat) and processed for sectioning. Sections (10 μm) were collected onto Fisher Superfrost Plus-coated slides, starting from where aorta exits the ventricle and moving towards the aortic sinus over ~650–700 μm. Sections were collected following the scheme as described (5), each slide (at least 5 sections per mouse) contained 9–12 aortic root sections collected at 40 μm intervals; with this scheme, consecutive, or immediately adjacent sections which are morphologically and compositionally identical, are located in separate slides, allowing for different staining methods to be conducted. Additional sections were collected at the end to be used as controls in immunostaining procedures. Lesion analysis with hematoxylin and eosin (H&E) staining was conducted as described previously (5). The images of lesions were taken with a Zeiss Axio Imager 2 microscope and analyzed by calculated percentages of aortic surface area covered by lesions using Image J software.

*Immunohistochemistry.* For immunostaining, cryosections on slides were fixed with cold acetone, permeabilized with 0.05% Triton X-100, and blocked with 5% normal goat serum. The sections were incubated with antibodies against Foxp3 (Thermo Fisher, 14-5773-82; 1:50) to detect Treg cells, CD68 (Bio-Rad, MCA1957; 1:500) to detect macrophages, CD206 (Bio-Rad, MCA2235; 1:500) to detect M2 macrophages, Ly5g+Ly5c (Abcam, ab25377; 1:100) to detect MDSCs, and arginase 1 (Thermo Fisher, PA5-85267; 1:100) to detect M2 macrophages and MDSCs. Sections were then incubated with appropriate secondary antibodies and detected with 3,3'-diaminobenzidine. The slides were counterstained with Mayer’s hematoxylin. To distinguish bona fide target staining from background, the secondary antibody only was used as a control. The images were taken with a Zeiss Axio Imager 2 microscope and analyzed using Image J software.

*Flow cytometric analysis.* To evaluate blood lymphocyte subsets, mouse blood samples were collected through submandibular vein puncture and incubated with ACK lysing buffer (Thermo Fisher, A1049201) at 37°C for 5 minutes. To evaluate aortic lymphocyte subsets, whole aortas were digested using a cocktail of Roche liberaseTH (4 U/ml) (Millipore/Sigma, 5401135001), deoxyribonuclease (DNase) I (0.1 mg/ml) (Millipore/Sigma, DN25), and hyaluronidase (60 U/ml) ((Millipore/Sigma, H3506) in 1 mol/L CaCl_2_ at 37°C for 15 min. The digested tissue was passed through a 70 μm cell strainer, washed with 1× cold PBS and centrifuged at 350*g* for 10 minutes at 4°C. Lymphocytes were first stained with live/dead cell fixable violet dead cell stain kit (Thermo Fisher, L34955; 1:100) and then blocked with TruStain FcX (anti-mouse CD16/32, BioLegend, 101320; 1:100). The cells were then stained with the following antibodies: anti-CD45 ̶ BV605 (BioLegend, 103140; 1:100), anti-CD3 ̶PE/Cy7 (BioLegend, 100220; 1:100), anti-CD8a ̶ PerCP/Cy5.5 (BioLegend, 100734; 1:100), anti-CD4 ̶ BV510 (BioLegend, 100559; 1:100), anti-NK1.1 ̶ APC (BioLegend, 108710; 1:100), anti-F4/80 ̶ APC/Cy7 (BioLegend, 123118; 1:100), anti-CD11b ̶ FITC (BioLegend, 101206; 1:100), anti-CD206 ̶ PE (BioLegend, 141704; 1:100), anti-CTLA-4 ̶ BV420 (BioLegend, 106311; 1:100), anti-CD39 ̶ APC (BioLegend, 143810; 1:100), anti-CD25 ̶ APC/Cy7 (BioLegend, 102026; 1:100), anti-Foxp3 ̶ PE (BioLegend, 126404; 1:50), anti-Gata3 ̶ AF488 (BD Bioscience, 560163; 1:50), anti-Tbet ̶ PerCP/Cy5.5 (BioLegend, 644806; 1:50), anti-RORgt ̶ APC (Thermo Fisher, 17-6981-82; 1:50), and anti-Ki67 ̶ AF700 (BioLegend, 652420; 1:50). For Gata3, RORgt, T-bet, Foxp3, and Ki67 staining, the cells were permeabilized using an Invitrogen^TM^ eBioscience^TM^ Foxp3 / Transcription Factor Staining Buffer Set (Thermo Fisher, 00-5523), and then stained with anti-Gata3, anti-RORgt, anti-Tbet, anti-Ki67, and anti-Foxp3 antibodies. Flow cytometry analysis was performed by FACSCelesta (BD Bioscience, Franklin Lakes, NJ, USA) and analyzed using FlowJo software. The antibodies used in this paper are listed in Supplementary Table 1.

*RNA isolation and quantitative PCR.* Total RNA was extracted from aortas after homogenized using the TRIzol reagent (Invitrogen, Carlsbad, CA, USA) and purified with RNeasy Mini Kit (Qiagen, Germantown, MD, USA). cDNAs were synthesized with the QuantiTect Reverse Transcription Kit (Qiagen, Germantown, MD, USA). Quantitative PCR (qPCR) was performed using a SsoAdvanced™ Universal SYBR® Green Supermix (BioRad, Hercules, CA, USA) and a QuantStudio 3 Real-Time PCR System (Applied Biosystems, Carlsbad, CA, USA) according to comparative threshold cycle method following manufacturer’s protocol. The amplification reactions were performed in duplicate, and the fluorescence curves were analyzed with the software included with the QuantStudio 3 Real-Time PCR System. 18s RNA was used as an endogenous control reference. The primers used for qPCR are listed in Supplementary Table 2.

*RNA-Seq analyses.* RNA-seq was performed by Genewiz/Azenta (South Plainfield, NJ, USA). Extracted RNA samples were quantified using a Qubit 2.0 Fluorometer (Life Technologies, Carlsbad, CA, USA) and RNA integrity was checked using an Agilent TapeStation 4200 (Agilent Technologies, Palo Alto, CA, USA). RNA sequencing libraries were prepared using the NEBNext Ultra II RNA Library Prep Kit for Illumina following manufacturer’s instructions (NEB, Ipswich, MA, USA). Briefly, mRNAs were first enriched with Oligo(dT) beads. Enriched mRNAs were fragmented for 15 minutes at 94 °C. First strand and second strand cDNAs were subsequently synthesized. cDNA fragments were end repaired and adenylated at 3’ ends, and universal adapters were ligated to cDNA fragments, followed by index addition and library enrichment by limited-cycle PCR. The sequencing libraries were validated on the Agilent TapeStation (Agilent Technologies, Palo Alto, CA, USA), and quantified by using a Qubit 2.0 Fluorometer (Invitrogen, Carlsbad, CA) as well as by quantitative PCR (KAPA Biosystems, Wilmington, MA, USA).

The sequencing libraries were clustered on 1 flowcell lane. After clustering, the flowcell was loaded on the Illumina HiSeq instrument (4000 or equivalent) according to manufacturer’s instructions. The samples were sequenced using a 2x150bp Paired End configuration. Image analysis and base calling were conducted by the HiSeq Control Software. Raw sequence data (.bcl files) generated from Illumina HiSeq was converted into fastq files and de-multiplexed using Illumina's bcl2fastq 2.17 software. One mismatch was allowed for index sequence identification.

*Data processing and visualization.* Sequence reads were trimmed to remove possible adapter sequences and nucleotides with poor quality using Trimmomatic v.0.36. The trimmed reads were mapped to the Mus musculus GRCm38 reference genome available on ENSEMBL using the STAR aligner v.2.5.2b. The STAR aligner is a splice aligner that detects splice junctions and incorporates them to help align the entire read sequences. BAM files were generated as a result of this step. Unique gene hit counts were calculated by using feature Counts from the Subread package v.1.5.2. The hit counts were summarized and reported using the gene-id feature in the annotation file. Only unique reads that fell within exon regions were counted. If a strand-specific library preparation was performed, the reads were strand-specifically counted. After extraction of gene hit counts, the gene hit counts table was used for downstream differential expression analysis. Using DESeq2, a comparison of gene expression between the control and HCW9302 groups of samples was performed. The Wald test was used to generate p-values and log2 fold changes. Genes with an adjusted p-value < 0.05 and absolute log2 fold change > 1 were called as differentially expressed genes for each comparison.

A gene ontology analysis was performed on the statistically significant set of genes by implementing the software GeneSCF v.1.1-p2. The mgi GO list was used to cluster the set of genes based on their biological processes and determine their statistical significance.

To estimate the expression levels of alternatively spliced transcripts, the splice variant hit counts were extracted from the RNA-seq reads mapped to the genome. Differentially spliced genes were identified for groups with more than one sample by testing for significant differences in read counts on exons (and junctions) of the genes using DEXSeq. For groups with only one sample, the exon hit count tables were provided. The read counts data were analyzed with GraphPad Prism 9. The RAN-seq data can be accessed at: https://doi.org/10.6084/m9.figshare.21718013.v1

*Statistics.* Statistical analyses were performed using GraphPad Prism 9. All numerical values are presented as mean values ± SEM. Statistical significance between groups was determined by 1-way ANOVA with Tukey’s correction or by unpaired 2-tailed t test. For each test, a *P* value of less than 0.05 was considered statistically significant.

*Study approval.* All animal studies were approved by the Institutional Animal Care and Use Committee of HCW Biologics, Inc.

**References**

1. Hughes MS, Yu YY, Dudley ME, Zheng Z, Robbins PF, Li Y, et al. Transfer of a Tcr Gene Derived from a Patient with a Marked Antitumor Response Conveys Highly Active T-Cell Effector Functions. *Hum Gene Ther* (2005) 16(4):457-72. doi: 10.1089/hum.2005.16.457.

2. Zhu X, Marcus WD, Xu W, Lee HI, Han K, Egan JO, et al. Novel Human Interleukin-15 Agonists. *J Immunol* (2009) 183(6):3598-607. Epub 20090826. doi: 10.4049/jimmunol.0901244.

3. Marín Morales JM, Münch N, Peter K, Freund D, Oelschlägel U, Hölig K, et al. Automated Clinical Grade Expansion of Regulatory T Cells in a Fully Closed System. *Front Immunol* (2019) 10:38. Epub 20190201. doi: 10.3389/fimmu.2019.00038.

4. Mohanta S, Yin C, Weber C, Hu D, Habenicht AJ. Aorta Atherosclerosis Lesion Analysis in Hyperlipidemic Mice. *Bio Protoc* (2016) 6(11). doi: 10.21769/bioprotoc.1833.

5. Daugherty A, Tall AR, Daemen M, Falk E, Fisher EA, Garcia-Cardena G, et al. Recommendation on Design, Execution, and Reporting of Animal Atherosclerosis Studies: A Scientific Statement from the American Heart Association. *Arterioscler Thromb Vasc Biol* (2017) 37(9):e131-e57. Epub 20170720. doi: 10.1161/ATV.0000000000000062.

**Supplementary Figure 1. Representative flow cytometry plots for Figure 4 Activation and expansion of Treg cells in HCW9302-treated *ApoE* deficient mice:** **(A)** CD4^+^CD25^+^Foxp3^+^ Treg cells, **(B)** NK1.1^+^CD3^-^ NK cells, **(C)** CD8^+^ T cells and CD4^+^ T cells, **(D)** CD4^+^Foxp3^+^ Treg and CD4^+^Foxp3^-^ Teff, **(E)** CD4^+^CD39^+^Foxp3^+^ Treg **(F)** CD4^+^CTLA4^+^Foxp3^+^ Treg.

**Supplementary Figure 2. Comparison of mouse body weight, plasma triglycerides, plasma LDL, and blood cytokines between control and HCW9302 treated *ApoE* deficient mice fed with WD: (A**) Mouse body weights, (**B**) plasma triglycerides, (**C)** plasma LDL, (**D**) IL-1β, (**E**) GM-CSF, (**F**) IL-6, (**G**) IL-10, (**H**) TNFα, (**I**) IL-12_p70_. Data are expressed as mean ± SEM (*n* = 9-10). Statistical analysis was performed using a 2-tailed, unpaired *t* test.

**Supplementary Figure 3. The mouse aortic lymphocyte subsets: (A**) CD4^+^ Th cells, (**B**) CD4^+^Tbet^+^ Th1 cells, (**C)** CD4^+^Gata3^+^ Th2 cells, (**D**) CD4^+^Rorgt^+^ Th17 cells were compared between control and HCW9302 treated *ApoE* deficient mice fed with WD at the end of study. Data are expressed as mean ± SEM (*n* = 8). Statistical analysis in **A-D** was performed using a 2-tailed, unpaired *t* test. **The** **representative flow cytometry plots**: (**E**) CD4^+^ Th cells, (**F**) CD4^+^Tbet^+^ Th1 cells, (**G**) CD4^+^Gata3^+^ Th2 cells, **(H)** CD4^+^Rorgt^+^ Th17 cells.

**Supplementary Figure 4. The mouse aortic lymphocyte subsets**: **(A**) CD8^+^ T cells, (**B**) NK1.1^+^CD3^-^ NK cells, (**C)** CD11b^+^F4/80^+^ macrophages were compared between control and HCW9302 treated *ApoE* deficient mice fed with WD at the end of study. Data are expressed as mean ± SEM (*n* = 8). Statistical analysis in **A-C** was performed using a 2-tailed, unpaired *t* test. **The** **representative flow cytometry plots**: (**D**) CD8^+^ T cells, (**E**) NK1.1^+^CD3^-^ NK cells, (**F**) CD11b^+^F4/80^+^ macrophages.

**Supplementary Table 1. Antibodies**

| Antibody | Conjugate | Clone | Species Reactivity | Host | Catalog# | Vender | Dilution |
| --- | --- | --- | --- | --- | --- | --- | --- |
| CD45 | BV605 | 30-F11 | Ms | mRt | 103140 | BioLegend, | 1:100 (FACS) |
| CD3 | PE/Cy7 | 17A2 | Ms | mRt | 100220 | BioLegend, | 1:100 (FACS) |
| CD8a | PerCP/Cy5.5 | 53-6.7 | Ms | mRt | 100734 | BioLegend, | 1:100 (FACS) |
| CD4 | BV510 | RM4-5 | Ms | mRt | 100559 | BioLegend, | 1:100 (FACS) |
| NK1.1 | APC | PK136 | Ms | mRt | 108710 | BioLegend, | 1:100 (FACS) |
| F4/80 | APC/Cy7 | BM8 | Ms | mRt | 123118 | BioLegend, | 1:100 (FACS) |
| CD11b | FITIC | M1/70 | Ms | mRt | 101206 | BioLegend, | 1:100 (FACS) |
| CD206 | PE | C068C2 | Ms | mRt | 141704 | BioLegend, | 1:100 (FACS) |
| CTLA-4 | BV420 | UC10-4B9 | Ms | mHm | 106311 | BioLegend, | 1:100 (FACS) |
| CD39 | APC | Duha59 | Ms | mRt | 143810 | BioLegend, | 1:100 (FACS) |
| CD25 | APC/Cy7 | PC61 | Ms | mRt | 102026 | BioLegend, | 1:100 (FACS) |
| Foxp3 | PE | MF-14 | Ms | mRt | 126404 | BioLegend, | 1:50 (FACS) |
| Gata3 | AF488 | L50-823 | Ms | mRt | 560163 | BD Biosciences | 1:50 (FACS) |
| Tbet | PerCP/Cy5.5 | 4B10 | Ms | mMs | 644806 | BioLegend, | 1:50 (FACS) |
| RORgt | APC | B2D | Ms | mRt | 17-6981-82 | Thermo Fisher | 1:50 (FACS) |
| Ki67 | AF700 | 16A8 | Ms | mRt | 652420 | BioLegend, | 1:50 (FACS) |
| CD8 | BV605 | SK1 | Hm | mMs | 344742 | BioLegend, | 1:50 (FACS) |
| CD25 | PE | M-A251 | Hm | mMs | 555432 | BD Biosciences | 1:100 (FACS) |
| CD4 | PerCP-Cy5.5 | RPA-T4 | Hm | mMs | 560650 | BD Biosciences | 1:50 (FACS) |
| CD56 | BV421 | 5.1H11 | Hm | mMs | 362552 | BioLegend, | 1:100 (FACS) |
| pSTAT5a | AF488 | 47/Stat5(pY694) | Hm | mMs | 612598 | BD Biosciences | 1:100 (FACS) |
| Foxp3 | unconjugated | FJK-16s | Ms | mRt | 14-5773-82 | Thermo Fisher | 1:50 (IHC) |
| CD68 | unconjugated | FA-11 | Ms | mRt | MCA1957 | Bio-Rad | 1:500 (IHC) |
| CD206 | unconjugated | MR5D3 | Ms | mRt | MCA2235 | Bio-Rad | 1:500 (IHC) |
| Ly6g+Ly6c | unconjugated | RB6-8C5 | Ms | mRt | ab25377 | Abcam | 1:100 (IHC) |
| Arginase 1 | unconjugated | N/A | Ms | pRb | PA5-85267 | Thermo Fisher | 1:100 (IHC) |
| CD25 | unconjugated | PC61.5.3 | Ms | mRt | TIB-222 | ATCC | 500μg (IP) |
| IL-2 | Biotinylated |  | Hu | pGt | BAF202 | R&D Systems | 200ng/mL (ELISA) |
| TF | unconjugated |  | Hu | mHu | HCW9101 | HCW Biologics | 5μg/mL (ELISA) |

**Supplementary Table 2. Primers**

| **Target** | **Forward primer sequence (5' ͢ 3')** | **Reverse primer sequence (5' ͢ 3')** |
| --- | --- | --- |
| *Cdkn1a* | CGGTGTCAGAGTCTAGGGGA | CGAAGTCAAAGTTCCACCGT |
| *Cdkn2a/p16^INK4a^* | CCCAACGCCCCGAACT | GCAGAAGAGCTGCTACGTGAA |
| *Cdkn2a/p19^ARF^* | GCCGCACCGGAATCCT | TTGAGCAGAAGAGCTGCTACGT |
| *Pai-1* | AGGATCGAGGTAAACGAGAGC | TTGGTTGAGGGAATCATTCAT |
| *Ccl2* | GCATCCACGTGTTGGCTCA | CTCCAGCCTACTCATTGGGATCA |
| *Tnfa* | GTCTACTGAACTTCGGGGTGAT | ATGATCTGAGTGTGAGGGTCTG |
| *Il6* | CAGAGGATACCACTCCCAAC | CAATCAGAATTGCCATTGCAC |
| *Inos* | GCAGAGATTGGAGGCCTTGTG | GGGTTGTTGCTGAACTTCCAGTC |
| *Trem2* | GACCTCTCCACCAGTTTCTCC | TACATGACACCCTCAAGGACTG |
| *Prf1* | GAGAAGACCTATCAGGACCA | AGCCTGTGGTAAGCATG |
| *Gzmb* | CCTCCTGCTACTGCTGAC | GTCAGCACAAAGTCCTCTC |
| *18S rRNA* | GTAACCCGTTGAACCCCATT | CCATCCAATCGGTAGTAGCG |

**Supplementary Table 3. RNA-seq data:** The hit counts from control and HCW9302 groups (n=3) were shown. Using DESeq2, a comparison of gene expression between the control and HCW9302 groups of samples was performed. The Wald test was used to generate p-values and log2 fold changes. Genes with an adjusted p-value < 0.05 and absolute log2 fold change > 1 were called as differentially expressed genes for each comparison.

| Gene.name | log2FC | pvalue | padj | Control-1 | Control-2 | Control-3 | HCW9302-1 | HCW9302-2 | HCW9302-3 |
| --- | --- | --- | --- | --- | --- | --- | --- | --- | --- |
| Calm4 | 4.6694 | 0.0019612 | 0.0352667 | 7.971614 | 17.74813 | 2.156689 | 4.4419659 | 282.479945 | 428.267748 |
| Calml3 | 3.3971 | 0.0009519 | 0.019683 | 98.31658 | 73.72299 | 14.01848 | 128.817011 | 954.421601 | 875.766863 |
| Cav3 | 2.2964 | 7.71E-18 | 7.63E-16 | 62.44431 | 56.65748 | 53.91721 | 208.772397 | 318.541215 | 319.536558 |
| Nqo1 | 1.5093 | 7.70E-07 | 3.33E-05 | 94.33077 | 89.42326 | 79.79748 | 171.015687 | 335.369807 | 244.090426 |
| Gstp2 | 1.351 | 5.12E-08 | 2.51E-06 | 112.9312 | 154.2722 | 116.4612 | 255.413039 | 359.410653 | 369.094311 |
| Gpc1 | 1.138 | 4.81E-07 | 2.15E-05 | 1090.783 | 621.8671 | 793.6614 | 1775.67587 | 1881.19623 | 1848.43023 |
| Mef2c | 1.1304 | 1.17E-06 | 4.94E-05 | 2325.054 | 1476.508 | 1608.89 | 3221.53577 | 4086.94389 | 4526.76791 |
| Mgst3 | 1.1274 | 1.70E-08 | 8.69E-07 | 640.3864 | 1022.565 | 906.8875 | 1858.96273 | 1829.50841 | 1935.71105 |
| Gsto1 | 1.0511 | 0.0002518 | 0.0064481 | 3669.6 | 2840.383 | 1791.13 | 4584.10881 | 6566.75719 | 6046.78556 |
| Prkaa2 | 1.0059 | 1.02E-09 | 5.82E-08 | 647.0294 | 910.6155 | 805.5232 | 1616.87559 | 1506.15903 | 1632.44719 |
| Mapk12 | 1.3003 | 1.42E-10 | 8.61E-09 | 472.9825 | 412.9853 | 382.8122 | 833.979098 | 1034.95844 | 1250.03885 |
| Bmpr1b | 1.14 | 6.21E-05 | 0.0018557 | 87.68776 | 63.48369 | 50.68218 | 133.258977 | 168.285925 | 140.536912 |
| Trim54 | 3.1536 | 9.26E-83 | 1.22E-79 | 136.846 | 154.9548 | 153.1249 | 1204.88325 | 1260.94239 | 1504.48463 |
| Trim63 | 2.9796 | 1.88E-23 | 2.53E-21 | 264.3919 | 132.4283 | 129.4013 | 1298.16454 | 1387.15684 | 1438.65418 |
| Trim55 | 2.1325 | 2.10E-13 | 1.55E-11 | 127.5458 | 68.26203 | 78.71913 | 342.031374 | 387.057627 | 460.07347 |
| Synm | 2.0166 | 7.62E-19 | 8.01E-17 | 632.4147 | 823.2401 | 719.2556 | 2184.33673 | 3014.72214 | 3608.1003 |
| Abcb4 | 1.9426 | 3.24E-27 | 5.45E-25 | 207.262 | 187.7206 | 210.2771 | 708.493561 | 697.184545 | 912.010593 |
| Mlip | 1.9188 | 9.35E-15 | 7.65E-13 | 126.2172 | 80.5492 | 73.32741 | 348.694323 | 342.582061 | 356.519956 |
| Dusp26 | 1.6414 | 3.89E-11 | 2.46E-09 | 67.75872 | 107.854 | 112.1478 | 277.622869 | 290.894241 | 335.809253 |
| Tead1 | 1.4401 | 1.69E-26 | 2.73E-24 | 732.0599 | 806.8572 | 823.855 | 1898.94042 | 2168.48434 | 2346.22677 |
| Fam134b | 1.4137 | 7.19E-26 | 1.11E-23 | 1496.006 | 1223.256 | 1191.57 | 3535.80486 | 3309.22251 | 3560.76155 |
| Synpo2 | 1.1794 | 3.97E-11 | 2.51E-09 | 1886.615 | 1446.472 | 1503.212 | 3064.95647 | 3856.15176 | 4023.05403 |
| Enho | 1.0734 | 0.000194 | 0.0051742 | 49.15829 | 77.81871 | 51.76053 | 115.491113 | 140.638951 | 125.743553 |
| Kmo | -1.042 | 0.002117 | 0.0375211 | 856.9486 | 373.3933 | 482.0199 | 229.871735 | 286.086072 | 313.619215 |
| Adamts4 | -1.368 | 3.80E-05 | 0.0012032 | 93.00217 | 68.26203 | 109.9911 | 32.2042528 | 32.4551426 | 39.2024018 |
| Gsdmc4 | -4.772 | 0.0004063 | 0.0097797 | 9.300217 | 9.556684 | 67.93569 | 2.22098295 | 1.20204232 | 0 |
| Serpinb1c | 4.5918 | 7.17E-14 | 5.47E-12 | 7.971614 | 3.413101 | 2.156689 | 59.9665397 | 121.406274 | 127.222889 |
| Mafa | 4.33 | 2.15E-09 | 1.18E-07 | 1.328602 | 8.874064 | 1.078344 | 59.9665397 | 80.5368354 | 113.169198 |
| Trim29 | 3.4044 | 1.63E-05 | 0.0005628 | 33.21506 | 19.79599 | 8.626754 | 36.6462187 | 394.269881 | 218.202048 |
| Trim72 | 3.3692 | 3.74E-109 | 1.38E-105 | 227.191 | 177.4813 | 216.7472 | 2288.72293 | 2008.61272 | 2078.46696 |
| Ybx3 | 1.5219 | 4.83E-17 | 4.63E-15 | 2549.588 | 3400.814 | 3579.025 | 8077.71499 | 9121.09712 | 10174.8724 |
| Ptgr1 | 1.3134 | 0.001685 | 0.0312272 | 235.1626 | 245.7433 | 207.0421 | 238.755667 | 776.519338 | 694.548213 |
| Cd7 | -1.029 | 0.0019424 | 0.0350572 | 179.3613 | 116.0455 | 111.0695 | 47.7511334 | 74.5266238 | 75.4461318 |
| Ccr6 | -1.26 | 0.0028345 | 0.0474189 | 534.0982 | 182.9422 | 272.8211 | 106.607182 | 165.88184 | 139.797244 |
| Itgb6 | 2.8438 | 5.27E-21 | 6.01E-19 | 38.52947 | 20.47861 | 24.80192 | 187.673059 | 213.963533 | 182.697986 |
| Neurl1a | 2.6911 | 3.25E-17 | 3.13E-15 | 81.04475 | 185.6727 | 186.5536 | 876.177774 | 1007.31146 | 1060.68385 |
| Vtcn1 | 1.6026 | 0.0009004 | 0.0188073 | 53.1441 | 27.98743 | 14.01848 | 92.1707925 | 106.981766 | 86.5411512 |
| Tgfb2 | 1.1741 | 8.65E-05 | 0.0025037 | 93.00217 | 128.3326 | 118.6179 | 172.126179 | 301.712622 | 295.867183 |
| Rnf128 | 1.0772 | 0.0002337 | 0.0060924 | 245.7914 | 240.2823 | 131.558 | 357.578255 | 454.371997 | 491.139525 |
| Bach2 | -1.111 | 0.0003476 | 0.0085442 | 872.8918 | 447.1163 | 580.1492 | 217.656329 | 320.945299 | 338.767925 |
| Ly6g6c | 4.0847 | 5.22E-06 | 0.0001935 | 7.971614 | 6.826203 | 2.156689 | 13.3258977 | 133.426697 | 140.536912 |
| Abca12 | 3.7685 | 8.62E-05 | 0.0024987 | 7.971614 | 4.095722 | 2.156689 | 7.77344033 | 80.5368354 | 101.33451 |
| Alox15 | 2.532 | 1.31E-05 | 0.0004616 | 106.2882 | 206.1513 | 99.20767 | 216.545838 | 923.168501 | 1246.34051 |
| Retnla | 1.1558 | 0.00024 | 0.0062214 | 872.8918 | 1512.687 | 1030.897 | 1704.60442 | 2509.86436 | 3401.73294 |
