# Supplementary figures and images for: A novel interleukin-2-based fusion molecule, HCW9302, differentially promotes regulatory T cell expansion to treat atherosclerosis in mice
Source: Front Immunol. 2023 Jan 25;14:1114802. doi: 10.3389/fimmu.2023.1114802 (PMC9907325; doi:10.3389/fimmu.2023.1114802)

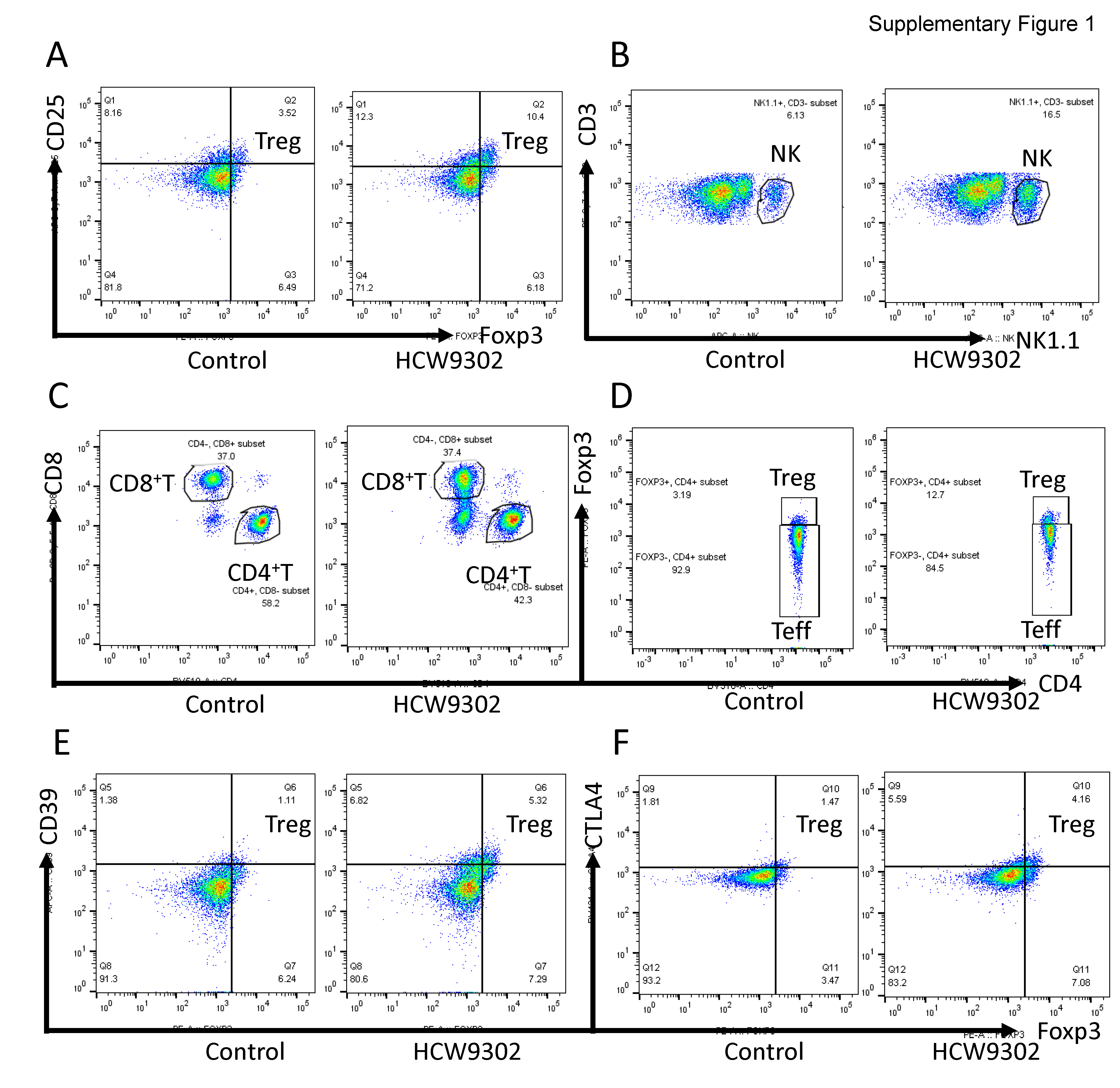

Supplement: Supplementary file 2 [file Image_1.tiff]

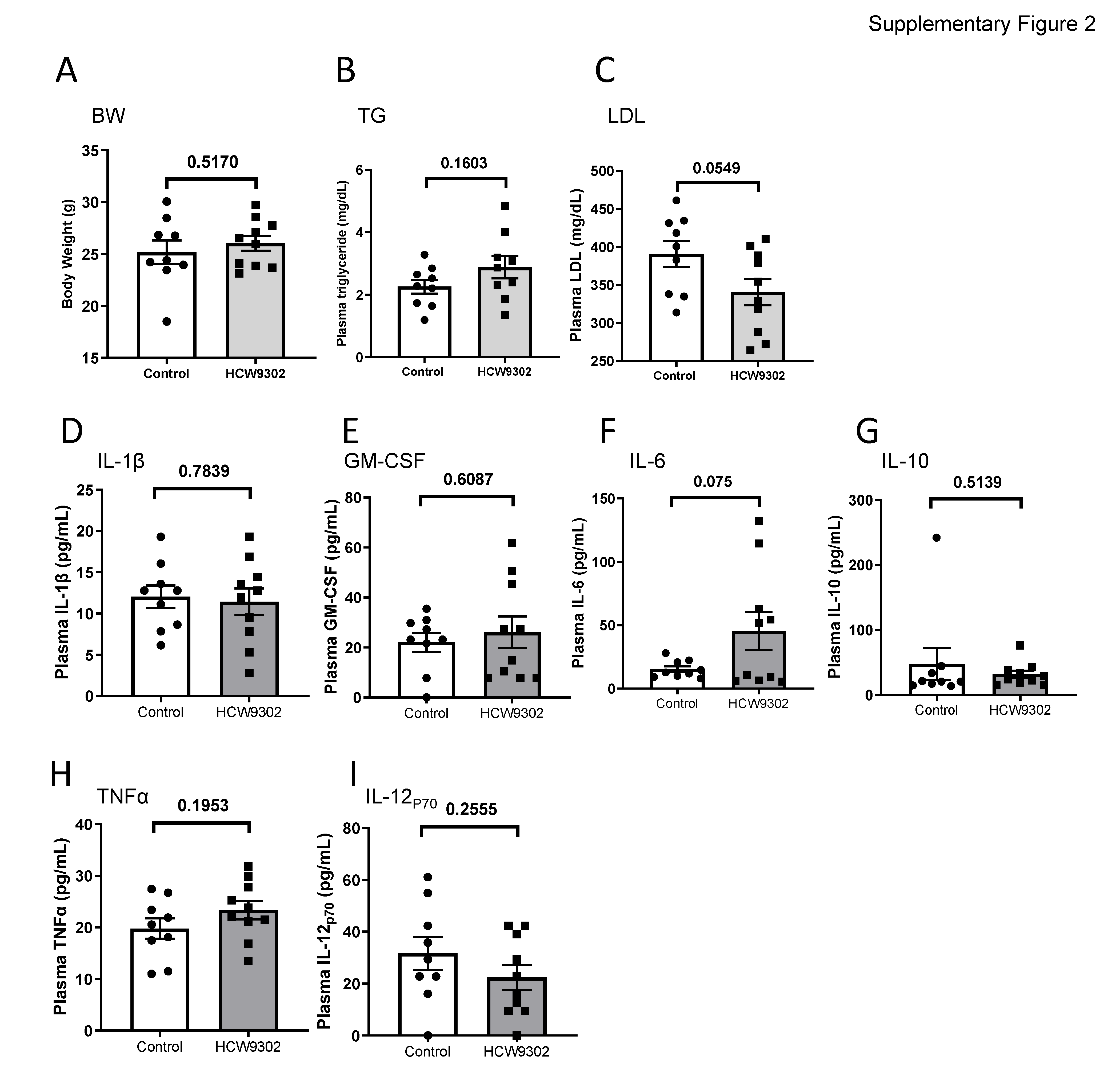

Supplement: Supplementary file 3 [file Image_2.tiff]

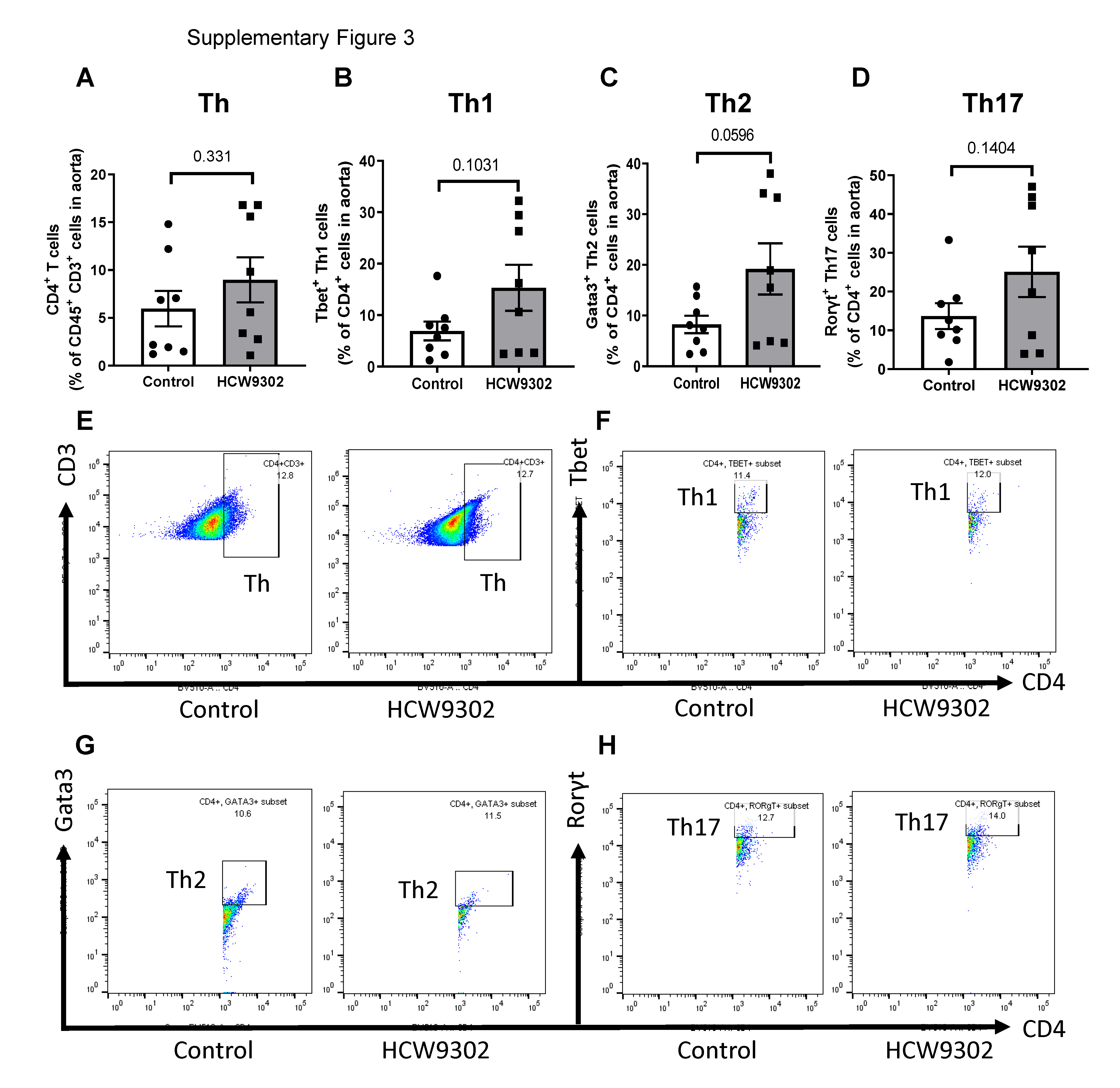

Supplement: Supplementary file 4 [file Image_3.tiff]

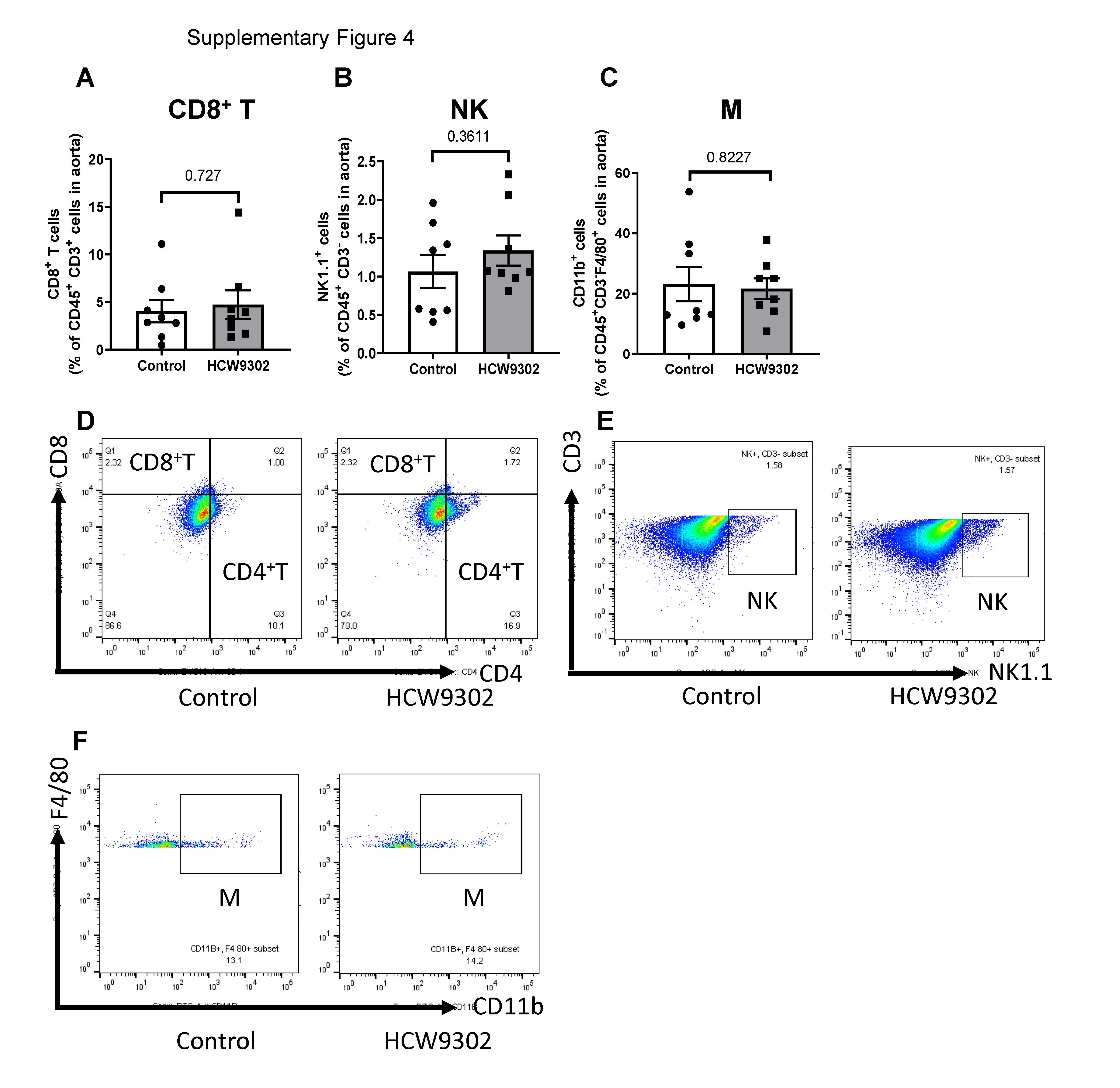

Supplement: Supplementary file 5 [file Image_4.tiff]
